# AGAPE (Computational G‑Quadruplex Stabilization Prediction): The First Machine Learning Workflow for G‑Quadruplex Stabilization Prediction

**DOI:** 10.1021/acsomega.6c03072

**Published:** 2026-05-21

**Authors:** Luisa D’Anna, Salvatore Contino, Rosalinda Marinello, Julie Fares, Giada De Simone, Antonio Monari, Florent Barbault, Giampaolo Barone, Alessio Terenzi, Ugo Perricone

**Affiliations:** † Department of Biological, Chemical and Pharmaceutical Sciences, 18998 University of Palermo, Viale Delle Scienze, Ed. 17, 90128 Palermo, Italy; ‡ Department of Engineering, University of Palermo, Viale Delle Scienze, 90133 Palermo, Italy; § 553061 Fondazione Ri.MED, Molecular Informatics Group, Corso Calatafimi 414, 90100 Palermo, Italy; ∥ 555089 Université Paris Cité and CNRS, ITODYS, F-75006 Paris, France

## Abstract

AGAPE (computational G-quadruplex stabilization prediction) is a novel machine learning (ML)-based tool designed to predict the stabilizing potential of small molecules targeting G-quadruplexes (G4s). G4s, prevalent in telomeres and oncogene promoters, are promising therapeutic targets, but designing selective binders remains challenging. Building upon a curated data set of 1217 compounds labeled through Förster Resonance Energy Transfer (FRET) melting assay data, AGAPE integrates 5666 molecular descriptors, both classical and quantum chemical. It captures features relevant to G4 recognition, driving researchers to predict the potential G4 stabilization of small molecules, including both organic ligands and metal complexes. Among the trained ML models, XGBoost achieved the best performance with an accuracy of nearly 91%, using 489 selected features. SHAP analysis highlighted descriptors related to molecular topology, polarizability, and electrostatic potential as key contributors to the classification. AGAPE is deployed through a user-friendly web interface, http://agape.fondazionerimed.com/, supporting batch prediction and secure data handling, and provides a robust and interpretable tool to accelerate the discovery of G4-stabilizing compounds, integrating quantum chemical information within an ML-driven cheminformatics framework.

## Graphical abstract

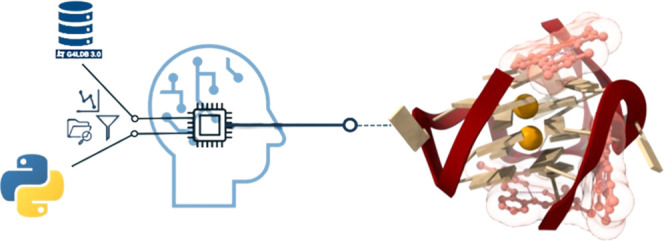

## Introduction

Guanine quadruplexes (G4s) are nucleic acid structures that play critical roles in diverse biological processes.^1^ In the past few years, these nucleic acid sequences have also become widely used in nanotechnology, for example, for drug delivery.^2^ They form in guanine-rich DNA and RNA sequences through Hoogsteen hydrogen bonding, resulting in stacked guanine tetrads stabilized by monovalent cations (e.g., Na^+^, K^+^, Figure 1),^3,4^ in some cases requiring concurrent coordination of K^+^ and Na^+^ ions at two distinct binding sites.^5^ Despite their rigid core, G4s exhibit remarkable structural polymorphism, with variations in the strand orientation, loop features, and glycosidic bond orientations, leading to parallel, antiparallel, and hybrid topologies. These polymorphic features influence the G4 stability and biological function.^1,6^

**Figure 1:**
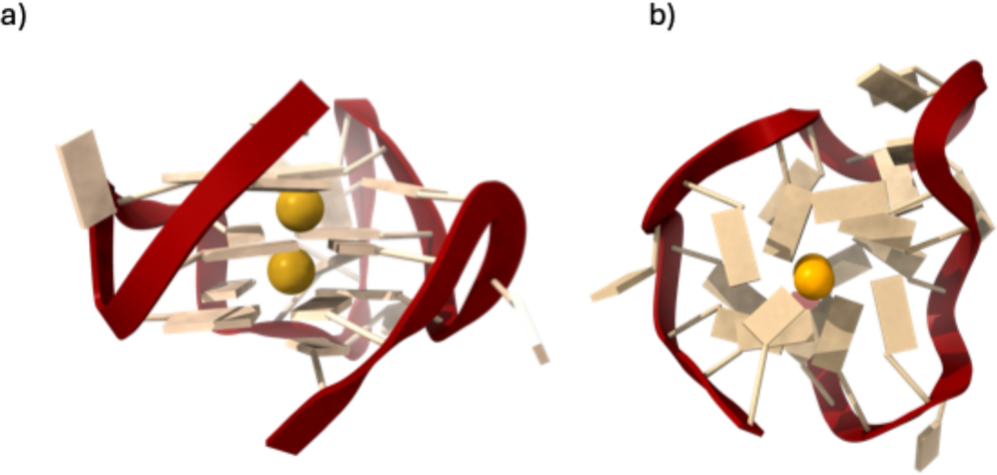
Side (a) and top (b) view of a typical parallel G-quadruplex motif.

G4 motifs are highly conserved in telomeres and oncogene promoters, where they regulate gene expression, replication, and chromosomal stability.^7,8^ Alterations in G4 stability have been linked to diseases such as cancer and neurodegenerative disorders, underscoring their potential as therapeutic targets.^9^ Small molecules designed to stabilize G4 structures typically feature extended aromatic scaffolds to promote stacking interactions with external G-quartets, as well as positively charged groups to enhance electrostatic interactions with the nucleic acid backbone.^10^ However, additional features are necessary to deploy stable interactions with the G4 loops, grooves, and extruded bases.^6^ Various organic and metal-based G4 binders have shown promising affinity and selectivity, also as fluorescent probes.^11–16^ Recently, a tetra-substituted naphthalene diimide targeting DNA G4s has recently advanced to a Phase I clinical trial for pancreatic ductal adenocarcinoma and other solid tumors.^17^ Notably, emerging evidence suggests that G4 ligands may also interact with additional cellular components and compartments, including lysosomes and mitochondria.^18^

Among metal-based G4 binders, our group synthesized different Salphen metal complexes that have attracted significant interest due to their strong and selective binding to G4 structures.^13,19–22^

However, challenges persist in achieving a high specificity for disease-relevant G4 structures. Ideal G4 binders should selectively recognize specific G4 motifs implicated in disease progression, modulating only associated biological pathways.

The selective targeting of G4s requires a deeper understanding of the structural features that govern G4–binder interactions. Current approaches often rely on molecular modeling, quantum chemical calculations, and experimental techniques such as calorimetry and spectroscopy. However, these methods are time-consuming and resource-intensive. Conversely, machine learning (ML) offers a transformative approach, enabling the rapid analysis of large data sets to predict molecular interactions and guide drug discovery.^23–25^ ML has already demonstrated success in predicting biological activities, physicochemical properties,^26–29^ and protein structures, as evidenced by the 2024 Nobel Prize in Chemistry awarded for advancements in computational protein design.^30^ Importantly, Schneekloth and colleagues developed a very important ML method for investigating RNA binders.^31^

Despite the growing application of AI in drug discovery, its use in designing selective G4 binders remains unexplored. Existing AI tools for G4 research primarily focus on predicting G4 folding and stability rather than host–guest binding.^32–37^ Gozalbes and co-workers have recently developed a computational tool for the screening of small-molecule ligands with the potential to target G4 DNA structures associated with cancer. This approach relies on multitask QSAR models built using both linear discriminant analysis and random forest machine learning algorithms.^38^

It is worth noting that the current data set of G4 binders is relatively limited compared to other biologically relevant targets, such as kinase inhibitors.^39,40^ Still, this relative scarcity of data and specialized tools underscores the urgent need to collect and organize information that can contribute to the building and feeding of an initial data-driven framework.

To address this gap, we developed AGAPE (computational G-quadruplex affinity prediction), the first ML-based framework designed to predict the G4 stabilization potential of small molecules, both organic and metal complexes. Beyond its predictive capabilities, AGAPE aims to elucidate the key chemical features that underpin G4 binding by using explainable AI techniques to enhance the model interpretability. Our approach utilizes molecular descriptors, including molecular embeddings,^41,42^ to construct a binary classification model that categorizes the compounds as active or inactive based on their G4 stabilization potential.

The use of molecular descriptors in ML applications is highly valuable across various domains^43–45^ as it facilitates the development of interpretable models compared to the use of molecular fingerprints for structural representation.^41,43,44^ Given the complex molecular and electronic nature of certain G4 binders, such as metal complexes, we have also incorporated quantum chemical (QC) properties as additional features to characterize these molecules. QC molecular descriptors provide detailed insights into molecular interactions, and their integration with ML has been proven useful and efficient in predicting physicochemical properties,^46–49^ reactivity, and^50^ regioselectivity of substitution,^51^ as well in the exploration of the chemical space.^52^ Moreover, combining QC descriptors with ML techniques shows significant promise in medicinal chemistry for drug design and lead discovery.

## Experimental Section

### Data Collection and Data Set Creation

Data were mainly retrieved from the public database G4LDB (v. 2.2),^53^ a collection aimed to explore molecules (mainly organic compounds) targeting all kinds of G-quadruplexes (DNA and RNA), as well as from compounds from the literature not included in G4LDB complemented by our in-house compounds library. To classify molecules in our data set as ACTIVE or INACTIVE, we relied on Förster Resonance Energy Transfer (FRET) melting assays, adopting thresholds informed by benchmark compounds from the G4LDB database and supporting literature reports.^53–58^ Specifically, molecules exhibiting Δ*T* ≤ 8 °C were labeled INACTIVE, whereas those with Δ*T* ≥ 15 °C were considered ACTIVE.

Although these cutoffs were inspired by literature precedents, the final thresholds were intentionally defined by us and explicitly stated in the usage guidelines of our tools to ensure transparency and reproducibility. Notably, while a Δ*T* of 15 °C is commonly regarded as indicative of significant G4 stabilization, we adopted a more conservative framework aimed at robustly identifying truly inactive compounds and reducing the likelihood of false positives.

The QC geometry optimization of the selected molecules was performed using the Jaguar tool from the Schrodinger suite (Schrodinger Release 2023–2: Jaguar, Schrodinger, LLC, New York, NY, 2024). Density functional theory (DFT) calculations, performed with B3LYP as the exchange–correlation functional and the LANLD2Z basis set, were used to optimize the molecular geometry and calculate all the relevant quantum chemistry (QC) properties. DFT calculations are indeed particularly useful to obtain the geometry of small molecules, especially transition metal complexes. In fact, the parametrization of classical force fields for transition-metal complexes is still not straightforward, and force–field parameters for some of the metals in our data set are either missing or not standardized. In addition, resorting to the QC approach allows us to explicitly include features directly related to the electronic structure of the studied molecule without relying on any empirical assumption. Thus, we calculated the QC molecular properties and retained them as additional features for our model creation. The selected QC descriptors were mainly focused on the determination of point charge distribution, polarizability, electrostatic potentials, and surface size. Starting from the QC optimized geometries, we have also calculated classical molecular descriptors using alvaDesc software.^59,60^

### Data Cleaning and Preparation

The data set has been processed into 3 consecutive steps, namely, data cleaning, data transformation, and dimensionality reduction.

Data cleaning: the data set was organized into columns representing individual variables. Columns with constant values, entirely missing values, or more than 20% of missing values were removed to improve the data quality.

Data transformation: missing values in numeric columns containing integers have been replaced with the median value, while those in columns containing double-precision floating data points were substituted with the mean value.

Dimensionality reduction: we calculated the Pearson correlation coefficient of 53 for each pair of columns as a measure of the correlation between the two variables. Variables with a high degree of correlation (threshold set at 0.9) were identified and subsequently removed, allowing us to significantly reduce redundancy in the data set.

After these three steps of cleaning and preparation, the residual data set consisted of 1723 features, which were then used as descriptors for building our ML model.

### Preprocessing

The features were normalized to the range [0, 1] using the MinMaxScaler estimator. This scaler transforms each feature individually to fit within the specified range, using a linear transformation. Note that MinMaxScaler does not mitigate the effect of outliers. To prevent overfitting, two validation strategies were adopted.

- A split of 80% training, 10% validation, and 10% test set was used.
- 10-Fold cross-validation.

### Feature Selection Algorithm

Feature selection methods were used to reduce the number of properties related to the G-quadruplex binding properties. Furthermore, the dimensionality reduction of the feature set allows the use of a smaller and more interpretable data set. The main categories of feature selection used in this work are filter, embedded, and wrapper.

The filter methodology was applied first. This approach evaluates each feature independently from the predictive model, filtering out the most irrelevant variables. It relies on statistical metrics that compute scores between each independent variable and the target. As a result, features that are redundant with respect to the target variable were excluded.

The main advantage of filter methods is their computational efficiency. However, they do not account for the interactions between features.

To reduce the dimensionality of the data set and highlight the most relevant variables for prediction, three filter techniques were applied.

### Mutual Information: Measures the Statistical Dependence between Each Feature and the Target

ANOVA (analysis of variance) evaluates the statistical significance of differences between groups.

Chi-square measures the statistical independence among categorical variables.

The practical implementation of these filter methods was performed by using the SelectKBest class from the Scikit-learn library. This class allows the automatic selection of the top k features based on the scores computed by the specified statistical scoring function, i.e., mutual information classification for Mutual Information, f classification for ANOVA, and chi2 for the Chi-square test.

The embedded approach integrates feature selection within the model training process. The classifier learns which features are relevant during the training phase. This technique offers a good trade-off between the computational cost and predictive performance.

On the basis of these results, random forest coupled with filter methods appeared to slightly outperform the other methods and has shown better performance stability independently from the filter method. Thus, we have also decided to evaluate the embedded feature selection capability of random forest by employing the feature importance measure provided by the ensemble module of scikit-learn.

The wrapper methodology explores combinations of features through a search strategy that considers the predictive power of subsets. While it captures feature interactions, it is computationally more expensive than the other methods.

Specifically, we used a sequential forward selection, which belongs to the category of “greedy” approaches. These strategies are used to reduce the initial d-dimensionality to a k - dimensional feature space where k < d. These approaches are based on the automatic selection of the k subset inherent to the identified task. This step aims to improve computational efficiency by reducing the generalization error of the model through the removal of irrelevant or noisy features.

Starting from a set of features Y = {y1, y2, yd}, the algorithm is initialized with an empty set ϕ so that k = 0 (where k is the size of the subset). The next phase involves the gradual inclusion of features as follows (eq 1)­

1 $$x^{+}=\arg\;\max J\left(Xk+x\right),where\;x\in Y-XkX_{k+1}=Xk+x+k=k+1$$

where *x* ^+^ is the feature that maximizes our criterion function, i.e. the feature associated with the best performance of the classifier when added to *Xk*.

The procedure continues until the criterion is satisfied or until k = p, i.e., the a priori defined maximum size of subset K. In our work, we have set a maximum *p* = 1723, i.e., the total number of features present in the initial data set, to ensure exploration of all the features’ space.

### Machine Learning Models

The following machine learning algorithms were adopted and trained: decision tree (DT), random forest (RF), Gaussian Naive Bayes (NB), support vector machine (SVM), and XGBoost.

DT is a supervised learning method used for classification. The goal is to create a model that predicts the value of a target variable by learning simple decision rules inferred from data features. It is a model that offers advantages in terms of its interpretation and simplicity.

Building upon the strategy exploited with decision trees, we extended our models to RF, a more advanced approach that aggregates the predictions of multiple decision trees to produce a more accurate and stable output. As an ensemble learning method, RF constructs a large number of trees during the training phase.^61^ Each tree performs repeated feature splitting until a specific condition is met, using the following splitting criterion (eq 2)­

2 $$Entropy=\sum_{i=1}^{C}-f_{i\log f_{i}}Gini\;Index=\sum_{i=1}^{C}f_{i}\left(1-f_{i}\right)$$

where C is the number of unique labels and fi is the frequency of label i at the node.

Naive Bayes (NB) is an algorithm based on the Bayes theorem that assumes that all the features are mutually independent. Specifically, Gaussian NB has been used, which is based on computing a normal distribution assuming that each likelihood (P­(xi|y)) follows a normal distribution for each xi with y, as expressed in eq 3).

3 $$P\left(x_{i}|y\right)=\frac{1}{\sigma \sqrt{2\pi }}e^{-{\left(x-\mu \right)}^{2}/2\sigma^{2}}$$

SVM is one of the most widely used and effective algorithms for classification and regression. It is based on the definition of hyperplanes to separate data into perfect groups. It is originally designed to separate classes that are easily dividable using linear kernels but can be extended to more complex data adopting nonlinear kernels. This strategy is called the kernel trick and aims to maximize classification capacity.^62^ Its linear application is shown in eq 4)­

4 $$w^{Τ}x+b\geq 0\;\text{for}d_{i}=+1w^{Τ}x+b<0\;\text{for}d_{i}=-1$$

where w is the weight vector, x is the input vector, and b is the bias.

To improve the performance, we also tested the kernel functions, as reported in Table 1.

**Table 1:** Kernel Used on the SVC Training Phase

Kernel	Formula
RBF*	*K*(*x*,*x* ^′^) = exp(-γ|*x*–*x* ^′^|^2^)
Sigmoid	*k*(*x*_*i*_,*x*_*j*_) = tan h(κ*x*_*i*_·*x*_*j*_ + *c*)

Finally, XGBoost^63^ is a boosting technique that creates a predictive model using additive decision trees. The prediction for an instance is given by ^*yi* = ^P^ *k* = 1^*K*^ *f*_*k*_(*x*_*i*_), with each *f*_*k*_ being a regression tree. The model optimizes an objective function by combining the empirical loss and a regularization term to penalize the model’s complexity. The gradients and Hessians of the loss function are used to calculate *j* for each leaf. To assess the benefit from a split, the predictive quality before and after dividing a node in two is calculated accounting for gradients, Hessians, and the γ penalty.

Each model was tested in a specific evaluation phase using the most commonly used state-of-the-art criteria for classification tasks. Specifically, we have calculated the accuracy, i.e., the percentage of correct predictions out of the total number of predictions, defined by eq 5

5 $$Accuracy=\frac{TP+TN}{TP+TN+FP+FN}$$

where TP are the true positives, TN are the true negatives, FP are the false positives, and FN are the false negatives.

Furthermore, we also checked the precision index that quantifies the true positive ratio among all positive predictions (eq 6)­

6 $$Precision=\frac{TP}{TP+FP}$$

and the Recall, or Sensitivity, index, which measures the ratio between the true positive predictions over the sum between true positives and false negatives (eq 7), thus giving a measure of the discriminative capacity of the model

7 $$Recall=\frac{TP}{TP+FN}$$

Finally, the F1-Score (eq 8), i.e., the harmonic mean of precision and recall, was considered since it provides an analytical compromise for extremely unbalanced data sets such as that under consideration

8 $$F_{1}=2\cdot \frac{Precision\cdot Recall}{Precision+Recall}$$

Indeed, considering its ability to evaluate highly unbalanced data sets, the F1 score was the most used metric for both feature selection and overall model performance.

As for feature selection, the number of retained features was varied from 50 to 300 in steps of 10, assessing the overall performance with F1-score.

Importantly, during the evaluation phase of the best model, accuracy was replaced by balanced accuracy (eq 9) to take into account class imbalance.

9 $$balanced\;accuracy=\frac{sensitivity+specificity}{2}$$

$$specificity=\frac{TN}{TN+FP}$$

### Web Interface

The development of the AGAPE web interface (http://agape.fondazionerimed.com/) was carried out using the server-side Django 5.2.3 (Python 3.13) framework, combined with standard front-end technologies such as HTML5, SCSS, JavaScript, and the Bootstrap 5.3.6 CSS framework.

As shown in Figure 2, the interface is divided into several sections accessible via a navigation menu: a home page presenting the AGAPE project, a section dedicated to submitting molecules and their descriptors, a section for displaying results, and a section devoted to feedback and contact information. In its current version, AGAPE relies exclusively on the input of precomputed classical and quantum chemical descriptors provided by the user through either manual entry or CSV file upload.

**Figure 2:**
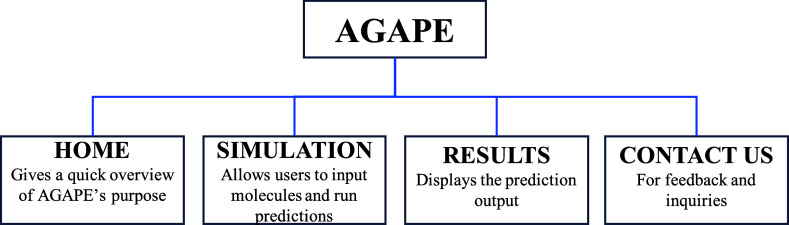
Overview of the AGAPE web interface, highlighting its different pages and their specific functions.

Supported descriptor formats include those generated by AlvaDesc (classical) and Jaguar (quantum). A downloadable example CSV file is available on the simulation page, illustrating the required column structure and formatting.

The schematic representation of the platform data flow is shown in Figure 3. Users submit input either manually or via a CSV upload. Classical and quantum chemical descriptors are merged and fed into the AGAPE machine-learning model, which predicts the likelihood of G4 stabilization by the given molecule.

**Figure 3:**
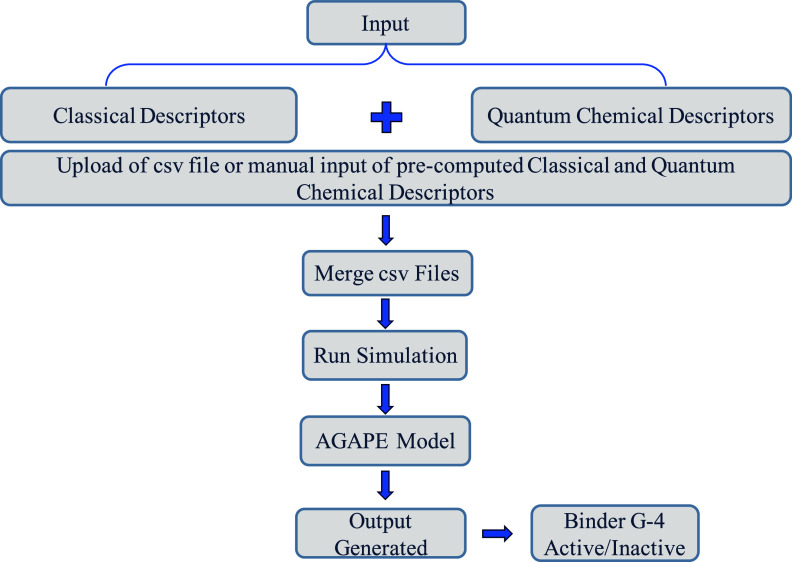
Overview of the platform’s data flow.

The results are displayed dynamically, accompanied by a classification message (ACTIVE/INACTIVE), and can also be downloaded in the CSV format. The web site allows batch processing, for over 1000 molecules in a single submission. Validation checkpoints have been implemented to control input formats, notably to ensure compliance with the input requirements defined by AGAPE and to prevent submission errors. The entire system was designed with ease of use and extensibility in mind.

The platform was deployed locally using Docker 28.2.2 and Docker Compose v2.37.1, encapsulating all dependencies into isolated containers to ensure environment reproducibility and portability. For production-level testing, the application was served using Nginx 1.28, a high-performance reverse proxy and web server, providing efficient static file delivery and routing. Hosting support was provided by Fondazione Ri.MED. The Ri.MED server infrastructure enables secure access for collaborative development and remote evaluation.

From a data privacy standpoint, no user data is stored permanently on the server. All files uploaded by users are temporarily processed during the session, strictly for the purpose of model inference, and are automatically deleted immediately after analysis. This ensures full compliance with data confidentiality best practices and minimizes the risk of a residual data exposure.

An upcoming version of the AGAPE platform aims to automate the generation of classical descriptors by using an integrated Python-based cheminformatics library. This functionality will be triggered either by direct submission of a SMILES string or via a molecule drawn using the JSME molecular editor, an interactive JavaScript-based tool embedded within the browser. The editor will automatically translate the drawn structure into its corresponding SMILES representation, which is then used to compute descriptors and feed them into the model. However, this automatization will require avoiding the use of commercial software for the prediction of the descriptors and will thus require retraining of the model.

## Results and Discussion

This section describes the results obtained during the different stages of our work. Specifically, the results obtained with the various feature selection methods are described, followed by the results obtained from training the best model for the selected feature subsets.

### Data Set Creation

The latest version of G4LDB includes 3695 G4 binders and 32142 activity entries. In Figure 4, a selection of well-known G4 binders is shown belonging to the mentioned database.

**Figure 4:**
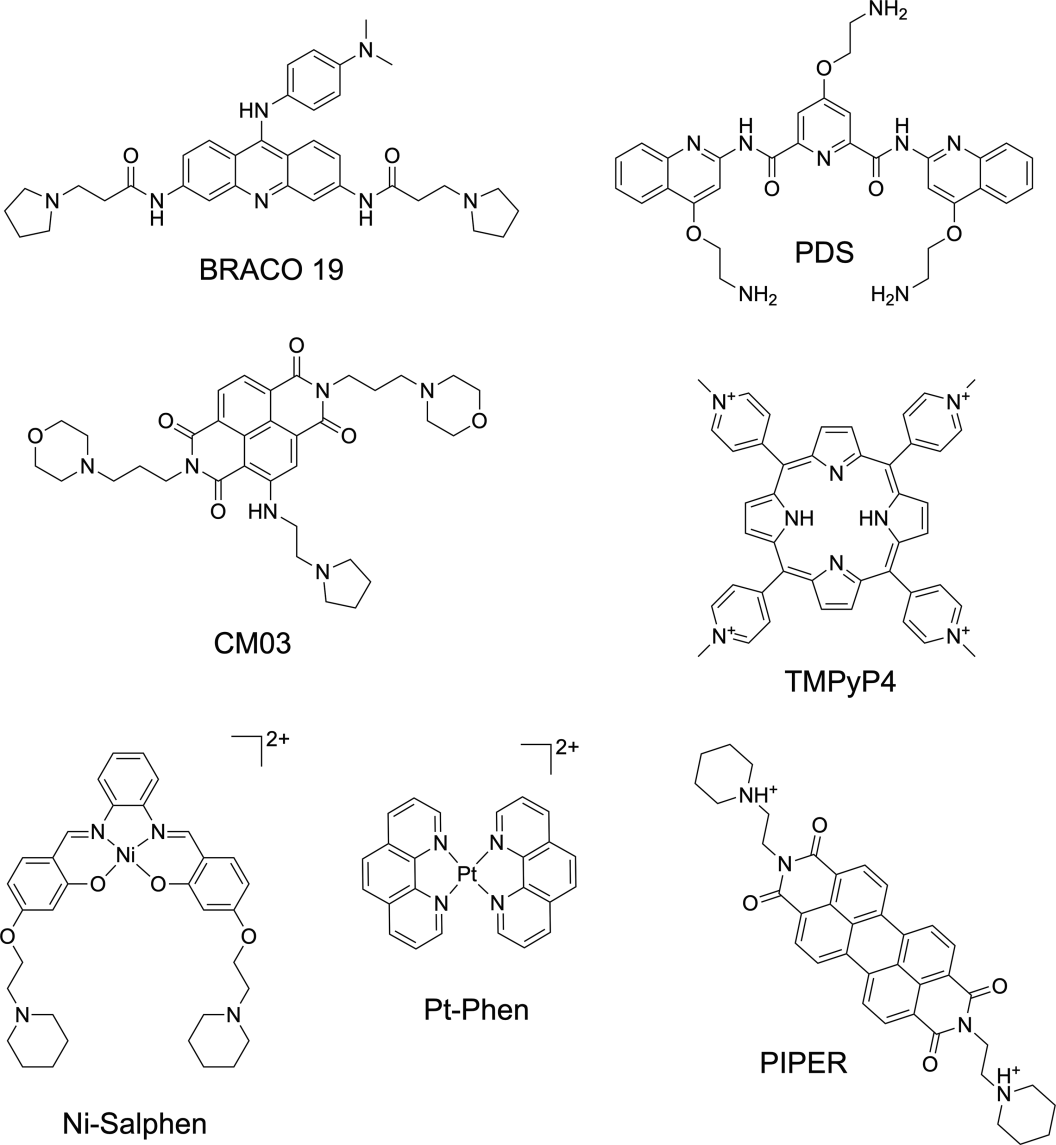
Representative list of well-known G4 binders included in the G4LDB database.

From this database, we focused on interaction and stabilization activities, specifically selecting data related to the FRET melting assay since the latter is a widely used technique to measure the melting temperature (*T*_m_) of G4 structures, providing insights into their thermal stability and the stabilization induced by small molecules.

In a typical FRET assay, a G4 sequence is labeled at its 5′ and 3′ ends with fluorescent donor and acceptor dyes. Upon excitation of the donor, energy transfer occurs if the acceptor is in close spatial proximity, thus quenching the donor fluorescence. At lower temperatures, G4s remain folded, keeping the donor and acceptor dyes close enough for FRET to occur. As the temperature rises, the G4 unfolds, increasing the distance between the dyes, reducing FRET efficiency, and enhancing the donor fluorescence quantum yield. In the presence of a stabilizing compound, the FRET assay is repeated. A stabilizer increases the *T*_m_ of the G4, indicating that the structure remains intact at higher temperatures. The shift in melting temperature (Δ*T*_m_) provides a direct measure of the stabilization induced by the binder. We selected this technique for its consistency with our in-house data set, which includes stabilization data obtained from FRET assays on various G4-DNA sequences. After filtering duplicates from G4LDB, we obtained 5320 unique activity entries and 1835 unique binders. Additional filtering ensured consistency of *T*_m_ data for molecules with multiple activity records. To align with our research group’s focus on metal complexes, particularly Salphen-based ligands known for their G4 stabilization activity,^13,20^ we supplemented the G4LDB collection with other Salphen-like complexes from literature sources.^64–67^

Ultimately, we compiled a unique data set comprising 1217 compounds, categorized into 490 ACTIVE and 727 INACTIVE entries. Among these, 1073 were purely organic compounds and 144 were metal complexes. Following QC geometry optimization and property calculations, the final data set included 1188 molecules.

### Descriptors Calculation

For each molecule, a total of 5666 molecular descriptors spanning 33 distinct classes were calculated using alvaDesc.^59^ These classes include connectivity indices, geometrical descriptors, 3D autocorrelations, functional group counts, charge descriptors, molecular properties, drug-like indices, and others. Detailed information about the descriptors can be found at https://www.alvascience.com/alvadesc-descriptors.

A preliminary dimensionality reduction step was performed to streamline the data set. Features with constant values, standard deviations below 0.0001, or columns containing all of the missing values were removed. This filtering process reduced the data set to 4285 conserved features from alvaDesc.

Additionally, relevant quantum chemical (QC) properties were computed through density functional theory (DFT) calculations. These included descriptors such as (i) surfaces: electrostatic potential, average local ionization energy, and electron densities; (ii) atomic electrostatic potential charges: charges and dipole moments; and (iii) electronic properties: Mulliken population, multipole moments, polarizability, and hyperpolarizability. The QC descriptors provide critical insights into molecular and electronic properties, complementing the alvaDesc features to enhance the predictive capabilities of the model. Moreover, they offer valuable information about metal complexes that are generally excluded from standard drug discovery workflows as their unique characteristics are not fully captured by traditional molecular descriptors.

### Feature Selection

#### Filter and Embedded FS

This section shows a summary table that collects the main results involving the selection of the most important features. Note that additional data are also presented in the Supporting Information (Tables S1–S11). Table 2 reports the top-performing model identified for each feature selection strategy used in the study using an 80:10:10 train–validation–test split. From these data, it appears that the most efficient model is RF combined with Mutual Information as the future selection method as it achieved the highest F1 score and, consequently, the highest accuracy. Table 3 summarizes the combinations of feature selection methods in cross-validation.

**Table 2:** Summary Table on the Test Set Split 80:10:10

Model	SS^a^	Accuracy	Precision	Recall	F1	# Features
DT	Mutual Info	0.8067	0.7551	0.7708	0.7629	140
DT	ANOVA	0.7983	0.7608	0.7291	0.7446	150
DT	Chi2	0.7899	0.7555	0.7083	0.7311	180
NB	Mutual Info	0.6891	0.6000	0.6875	0.6408	100
NB	ANOVA	0.6387	0.5490	0.5833	0.5657	190
NB	Chi2	0.6891	0.5846	0.7917	0.6726	210
RF	Mutual Info	0.8319	0.8333	0.7292	0.7778	260
RF	ANOVA	0.8151	0.7955	0.7292	0.7609	60
RF	Chi2	0.8319	0.8500	0.7083	0.7727	70
RF	RFI^b^	0.8151	0.8250	0.6875	0.7500	260

a SS = selection strategy.

b RFI = random forest importance.

**Table 3:** Summary Table on Cross-Validation

Model	SS^a^	Accuracy	Precision	Recall	F1	# Features
DT	Mutual Info	0.7972	0.7529	0.7428	0.7453	120
DT	ANOVA	0.7937	0.7530	0.7238	0.7352	240
DT	Chi2	0.7862	0.7217	0.7564	0.7368	130
NB	Mutual Info	0.6826	0.5984	0.6549	0.6196	160
NB	ANOVA	0.6919	0.6112	0.6461	0.6231	150
NB	Chi2	0.6170	0.5200	0.8573	0.6421	290
RF	Mutual Info	0.8636	0.8432	0.8081	0.8237	300
RF	ANOVA	0.8586	0.8473	0.7908	0.8150	290
RF	Chi2	0.8611	0.8400	0.8044	0.8204	60
RF	RFI^b^	0.8636	0.8470	0.8054	0.8236	100

a SS = Selection Strategy.

b RFI = Random Forest Importance.

The results highlight that RF consistently achieved better performance, reaching an F1 score of 0.8236 when using the embedded method.

#### Wrapper FS

As described in the Experimental Section, the selection of features with wrapper methods was conducted using Sequential Forward Selection with a maximum number of features of 1723. Indeed, to perform an exhaustive search of the features, it was essential to search within the entire d-dimensional space. Moreover, to ensure that all possible data distributions were tested, the cross-validation parameter (cv = 10) were set. In this way, feature selection was done using the average across folds to obtain a robust result that was invariant to different distributions. Afterward, once the parameters of the SFS algorithm were set, all the selected ML models have been tested. The results are shown in Figure 5.

**Figure 5:**
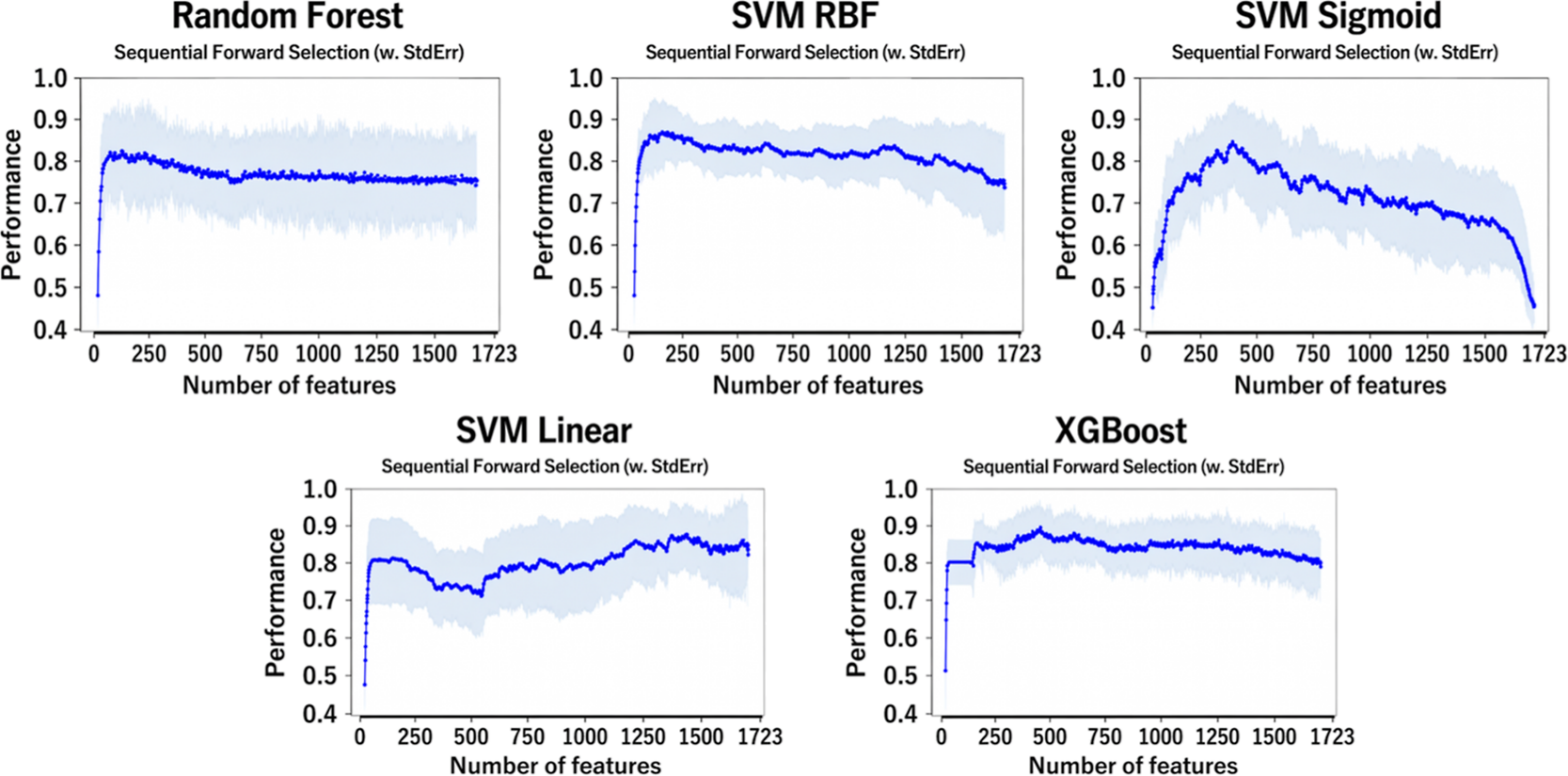
Plot showing comparative results obtained with various ML approaches in Sequential Feature Selection (SFS) on the total feature set.

The overall trend observed in the various feature selection studies is consistent with what has been reported in the literature. Indeed, the increase in performance is not directly related to the number of features utilized. However, as shown in Figure 5, it decreases for almost all models as the number of features increases. The best stability was obtained with the XGBoost model, which follows the trend discussed above but achieves higher performance in terms of F1-score than the other models. As can be seen from Figure 6, a peak performance of ∼ 0.93 is achieved.

**Figure 6:**
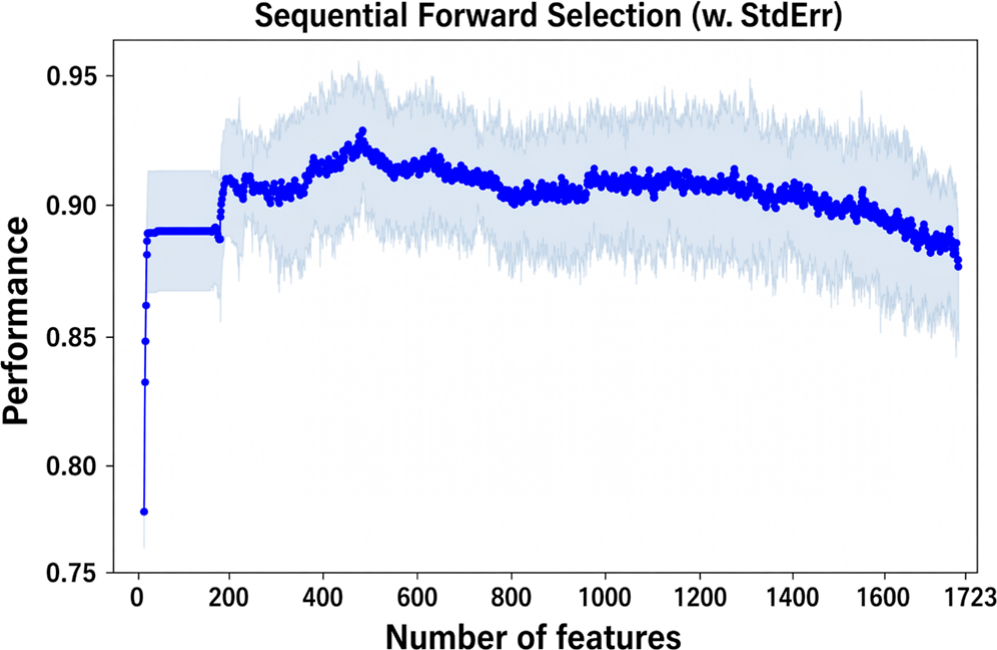
Plot of the SFS procedure on XGBoost.

XGBoost outperforms the models’ results regardless of the type of feature selection used. The highest performance was achieved using a collection of 489 features, which reduced the data set’s dimensionality by one-third. To further minimize data complexity, an additional subset of 180 features (one-tenth of the total) was selected, still yielding acceptable F1-scores. These two features set were then used to carry out the classification tests described in the following section.

### Classification

The classification trials were carried out using XGBoost in its best configuration, using the two subsets of features identified in the previous stages. To maximize the performance of XGBoost, hyperparameter tuning was performed by evaluating several parameter combinations. The optimum configuration was determined and is reported in Table 4.

**Table 4:** Summary of the Best Parameters Used for Training XGBoost

Parameter	Values	Description
n estimators	1000	Total number of trees (boosting rounds). A high value allows for more gradual learning, which is useful with a low eta
max depth	8	Maximum depth of trees. Higher values allow for more complex models but increase the risk of overfitting
eval metric	auc	Evaluation metric: AUC (Area Under the Curve), useful for evaluating the discriminatory capacity of the model
Booster	dart	Type of the booster used. dart (Dropouts meet Multiple Additive Regression Trees) is a boosting method that adds dropout to trees during training to reduce overfitting and improve generalization.
Eta	0.05	Learning rate
subsample	1	Percentage of samples used for each tree. One means that all data are used (no subsampling)
scale pos weight	3	Weight assigned to the positive class. Useful for managing unbalanced classes

The trials used 10-fold cross-validation to assess the algorithm’s robustness. Tables 5 and 6 show the results obtained for the two feature subsets (489 and 180) for all 10 folds; both tables indicate the average performance over the 10 folds evaluated on the last row.

**Table 5:** Results Obtained with XGBoost on a Larger (489) Subset of Features

Fold	F1	Bal. Acc.	Precision	Recall
1	0.9116	0.8802	0.8933	0.9306
2	0.9241	0.9014	0.9178	0.9306
3	0.9091	0.8848	0.9028	0.9155
4	0.9091	0.8575	0.8434	0.9859
5	0.9020	0.8505	0.8415	0.9718
6	0.8767	0.8361	0.8533	0.9014
7	0.9577	0.9476	0.9577	0.9577
8	0.9116	0.8781	0.8816	0.9437
9	0.8874	0.8335	0.8375	0.9437
10	0.8844	0.8407	0.8553	0.9155
	Avg F1	Avg Bal. Acc.	Avg Precision	Avg Recall
	0.9074	0.8711	0.8784	0.9396

**Table 6:** Results Obtained with XGBoost on a Smaller (180) Subset of Features

Fold	F1	Bal. Acc.	Precision	Recall
1	0.8811	0.8524	0.8873	0.8750
2	0.9333	0.9010	0.8974	0.9722
3	0.9209	0.9090	0.9412	0.9014
4	0.8961	0.8401	0.8313	0.9718
5	0.8774	0.8122	0.8095	0.9577
6	0.8611	0.8220	0.8493	0.8732
7	0.9379	0.9164	0.9189	0.9577
8	0.8811	0.8499	0.8750	0.8873
9	0.8552	0.8090	0.8378	0.8732
10	0.8732	0.8409	0.8732	0.8732
	Avg F1	Avg Bal. Acc.	Avg Precision	Avg Recall
	0.8917	0.8553	0.8721	0.9143

As shown from the average performances reported in Tables 5 and 6, both subsets consistently yielded good performances for each of the 10 folds calculated. The use of a higher number of features led to a peak sensitivity of 0.9396, proving the model’s ability to correctly discriminate truly active molecules. The performance is encouraging, especially considering the small number of training samples, even though they were tested exhaustively using 10-fold cross-validation.

To assess in more depth the accuracy of the model, an additional test data set consisting of 27 molecules (10 ACTIVE and 17 INACTIVE) was selected and used to validate the best algorithm (Table S12). While we acknowledge that a set of 27 molecules represents a relatively small validation cohort, it is important to emphasize that these compounds are fully independent and structurally uncorrelated with those included in the original data set. Notably, this external test set is enriched in metal-based complexes, a class that is underrepresented in the training data and contains a predominance of active compounds. We recognize that the composition of this validation set could be further improved by including a more balanced distribution of active and inactive compounds, better reflecting the proportions used during the model training. However, generating such a data set would require substantial additional experimental effort. The results obtained from both subsets are listed in Table 7. On the test set, the performance of our model drops. The cause behind this lack of performance could be ascribed to different reasons, not necessarily connected to the model effectiveness but rather due to statistical and sampling limitations inherent in small data sets used as an external set. In this case, our external set mainly contains inorganic compounds, thus leading to a biased estimate of the model generalization. Indeed, the model may analyze unfamiliar patterns or chemotypes, probably without having enough context to generalize, leading to degraded performance.

**Table 7:** Results Obtained on the Test Data

# Features	F1	Bal. Acc.	Precision	Recall	AUC
489	0.7357	0.6661	0.7832	0.7222	0.6661
180	0.6928	0.6644	0.6972	0.6889	0.6644

#### SHAP Analysis

SHAP^68^ is an Explainable AI approach based on game theory to explain the output and mechanisms underlying the prediction of a machine learning model. Specifically, SHAP computes the Shapley values for a given instance according to eq 10

10 $$g\left(z^{\prime }\right)=\phi_{0}+\sum_{j=1}^{M}\phi_{j}z_{j}^{\prime }$$

where *g* is the explanation of the model, *M* is the maximum simplified input features size contained in *z* ^′^, and ϕ_*i*_ ∈ R.

In our case, we used a variant of the SHAP explainer, namely, TreeExplainer,^69^ which is designed to interpret tree-based ML models such as decision tree, random forest, and gradient-boosted trees.

The results obtained from this analysis are shown in Figure 7, which displays a violin plot of the top 10 most relevant features.

**Figure 7:**
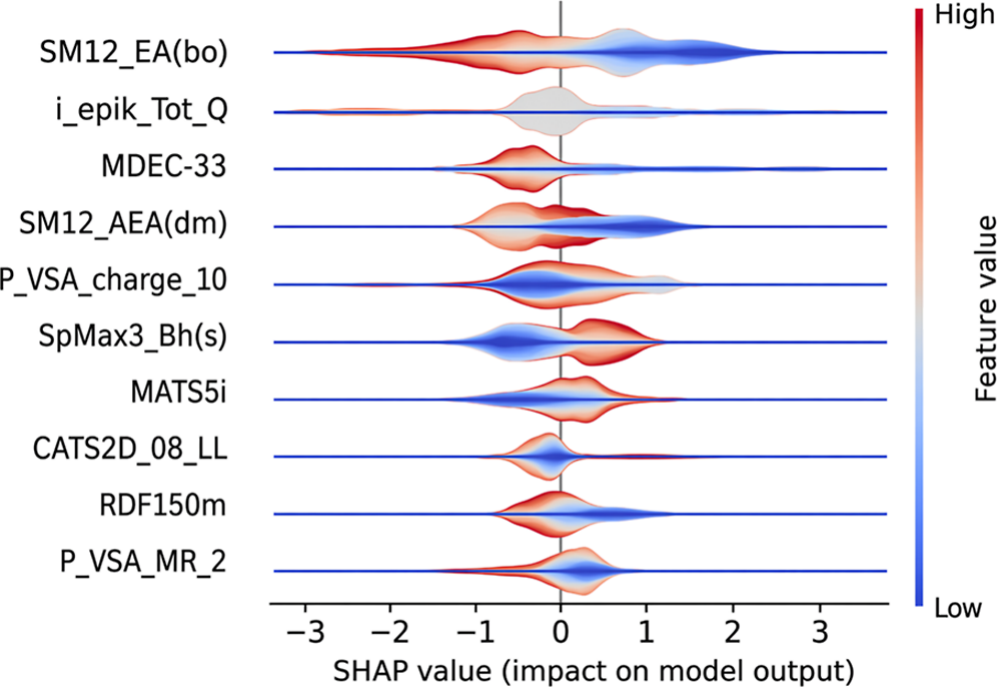
Violin plot that reports the contribution of the different features obtained with the SHAP analysis.

The relevance of each individual feature can be interpreted by evaluating the value of the individual Shapley values obtained. Specifically, values less than 0 indicate a negative impact on the prediction, while values greater than 0 suggest a positive contribution. Based on this assumption, we can see that feature SM12 EA (bo) (spectral moment of order 12 from edge adjacency mat. weighted by bond order) tends to negatively influence the correct classification when it assumes high values. This descriptor considers long-range interactions within molecular topology and it could reflect potential molecular rigidity and extensive conjugation. A very high value of this descriptor could refer to molecular complexity and rigidity, which might hinder the molecule’s ability to adopt an effective conformation to bind and stabilize a G4 conformation through π–π stacking or groove binding in G4 DNA. Molecules with excessively complex or branched topologies could indeed present steric hindrance or lack the surface planarity required for optimal binding.

In contrast, SM12 AEA­(dm) (spectral moment of order 12 from augmented edge adjacency mat. weighted by dipole moment) and SpMax3 Bh(s) (largest eigenvalue n. Three of Burden matrix weighted by I-state) are among those that positively influence classification performance when their values are high. SM12 AEA­(dm) is a variation of the previous descriptor, though considering polarity distribution into the topological structure. A favorable polar distribution across the molecular surface is crucial to stabilize the G4 conformation, and the polarity of the molecule could facilitate the binding process through polar interactions (i.e., hydrogen-bonding interactions). SpMax3 Bh(s) reflects electronic distribution and atomic hybridization within the molecule. High values of this descriptor could be related with delocalized π-electrons (aromatic systems or π conjugation), a favorable molecular characteristic to stabilize the DNA G4 conformation through π–π interactions.

Due to issues with the determination of molecular descriptors, the iepik Tot Q (net charge of the molecule) feature was set to 0 for all the compounds. Despite the fact that positive charge is an important parameter for inducing G4 stabilization, the model remains sufficiently robust to predict ligand behavior even in the absence of this parameter. We would also like to underline that in the training, test, and validation sets, no negatively charged compounds were represented. This is consistent with the well-established understanding that negatively charged ligands are unlikely to form stable complexes with G-quadruplexes due to unfavorable electrostatic repulsion with the nucleic acid phosphate backbone.

As shown by our results, the most influential features in distinguishing active from inactive molecules are those related to molecular topology, molecular polarity, and π conjugation, particularly the edge adjacency indices and Burden eigenvalues. Both descriptors effectively capture the global structure of the molecule while incorporating information about its electronic properties.^63,70,71^

Further building on the interpretation of the SHAP analysis, the identified descriptors can be translated into practical guidelines to support the rational design of new G4 ligands.

The behavior of SM12 EA (bo), a descriptor reflecting long-range topological interactions weighted by bond order, suggests that excessive molecular complexity may be unfavorable for the G4 binding process. As a matter of fact, molecules that are too complex or conformationally constrained may struggle to adopt the optimal geometry required for effective interaction with G-quartets or grooves. From a design perspective, it could be suggested that one should avoid overly bulky or highly branched scaffolds, instead preferring more compact structures with a certain degree of conformational adaptability.

SM12 AEA (dm) captures how dipole-related properties are spread across molecular topology. This suggests that a well-balanced and accessible polar profile can enhance interactions with G4 structures. In practical terms, this can be achieved by introducing strategically positioned heteroatoms or functional groups capable of hydrogen bonding, particularly in regions of the molecule that are likely to interact with groove or loop domains.

A similar trend is observed for SpMax3 Bh(s), which is associated with electronic distribution and atomic hybridization. Higher values are typical of extended π-conjugation and aromatic moieties, both of which are essential for stabilizing G4 DNA through π–π stacking interactions with the G-tetrads. This finding reinforces that planar, π-rich systems represent a favorable structural motif as they can efficiently stack onto the terminal tetrads and contribute to overall stabilization.

Taken together, these observations outline a coherent and intuitive design strategy: effective G4 binders should combine a sufficiently planar and conjugated core, a well-distributed polarity enabling secondary interactions, and a controlled level of structural complexity that avoids steric hindrance while preserving flexibility.

This kind of descriptor is particularly used for QSAR application and property prediction of small molecules because it considers the whole molecular connectivity and atomic feature at the same time. AGAPE represents, to our knowledge, the first ML-based framework for G4 stabilization that combines classical cheminformatics descriptors with quantum chemical properties and is implemented as an accessible Web-based tool. This proof of concept demonstrates the feasibility and added value of QM-informed descriptors in predictive modeling and provides a solid methodological basis for the development of more selective sequence-resolved models.

## Conclusions

In this work, we introduced AGAPE (computational G-quadruplex affinity prediction), the first in silico platform designed to predict the stabilization capacity of G4 binders. AGAPE employs a machine learning framework based on supervised classification, using molecular descriptors and quantum-chemical-derived properties of small molecules. To develop and train the models, we curated a data set of 476 ACTIVE and 712 INACTIVE compounds, enabling the identification of chemical features critical to G4 stabilization.

Among the tested classifiers, XGBoost demonstrated the best predictive performance, achieving an average F1 score of 0.91 and a peak sensitivity of 0.94 in cross-validation, confirming its strong generalization ability even with a limited number of training samples. The robustness of the model was further validated on an independent in-house library of 27 compounds, which included 24 transition-metal complexes and 3 organic ligands not included in the training, test, or validation sets. Despite the inorganic compounds representing the minority class in the original data set, the model achieved an overall accuracy of 66%. The deployment of AGAPE to screen the public database could also be envisaged to pinpoint relevant active organic G4 binders that could be prepared and tested to increase the representativity and statistical significance of the additional test set. Notably, feature importance analysis using SHAP provided interpretable insights, confirming the relevance of molecular topology and electronic structure descriptors, particularly edge adjacency indices and Burden eigenvalues, in determining the G4 binding potential.

The AGAPE framework is deployed through an accessible and secure web interface (http://agape.fondazionerimed.com/), allowing researchers to perform batch predictions based on user-supplied molecular descriptors. This platform not only enables high-throughput screening of potential G4 binders but also lays the foundation for integrating cheminformatics and quantum-informed descriptors in predictive modeling. We acknowledge that the present implementation of AGAPE considers all G-quadruplex sequences and topologies as a single predictive class, without explicitly incorporating sequence-dependent structural variability. While this represents a current limitation, it does not detract from the conceptual and practical significance of this work. To the best of our knowledge, AGAPE is the first machine learning-based platform for G4 stabilization that integrates classical molecular descriptors with quantum chemical features and is made available through an accessible online interface for the scientific community. By combining interpretability, QM-informed modeling, and user-oriented deployment, AGAPE establishes a new framework for predictive G4 ligand discovery and provides a foundation for the next generation of sequence-aware models.

Overall, AGAPE offers a novel, interpretable, and practical tool for accelerating the discovery of selective G4-targeting compounds. Future work will focus on expanding the data set and automating descriptor calculation from SMILES input. Probably the biggest limitation of the current version of AGAPE is the fact that our model is blind to any particular structural feature of the G4. However, our chosen data set is labeled with stabilization data of parallel, antiparallel, and hybrid G4s, without distinction. Including G4 structural information will be challenging, partially because of the lack of homogeneous experimental data and because of the need to combine features referring both to the ligands and G4 structures in the same model. Nevertheless, we aim to refine AGAPE, expanding the model to predict selectivity for G4 structures over canonical nucleic acids, as well as selectivity across different G4 topologies or sequences, which represents an exciting direction for further development. Additionally, integrating AGAPE with generative AI frameworks could enable the suggestion of structural modifications to existing G4 stabilizers or the design of novel scaffolds. By virtually labeling large data sets as ACTIVE or INACTIVE for G4 stabilization, AGAPE could provide the foundation for training generative AI or large language models (LLMs). These tools could mitigate the scarcity of experimental data and drive the discovery of new selective G4-targeting compounds.

WebApp address: http://agape.fondazionerimed.com.

Data set and ML models are available at https://github.com/Molinf-RiMED/AGAPE.

10.5281/zenodo.17277995.

## Supplementary Material



## Notes

Tables reporting the performance of the random forest, decision tree, and naive Bayes models with mutual information, ANOVA, Chi2, and random forest importance selection on different numbers of features; summary of feature selection using cross-validation; and structures of the 27 molecules from our in-house database (PDF)
