# Supplementary material for: AGAPE (Computational G‑Quadruplex Stabilization Prediction): The First Machine Learning Workflow for G‑Quadruplex Stabilization Prediction
Source: ACS Omega. 2026 May 21;11(21):31744–56. doi: 10.1021/acsomega.6c03072 (PMC13234782; doi:10.1021/acsomega.6c03072)
Supplement: Supplementary file 1 [file ao6c03072_si_001.pdf]

AGAPE (computAtional G-quadruplex stAbilization PrEdiction): The first Machine Learning workflow for G-quadruplex stabilization prediction

Luisa D'Anna [1‡], Salvatore Contino [‡2], Rosalinda Marinello [3], Julie Fares [4], Giada De Simone [3], Antonio Monari [4], Florent Barbault [4], Giampaolo Barone [1], Alessio Terenzi [1*], Ugo Perricone [3*]*

[1] *Department of Biological, Chemical and Pharmaceutical Sciences, University of Palermo, Viale delle Scienze, Ed. 17, 90128, Palermo, Italy.*
[2] *Department of Engineering, University of Palermo, Viale delle Scienze, 90133, Palermo, Italy.*
[3] *Fondazione Ri.MED, Molecular Informatics Group, Corso Calatafimi 414, 90100, Palermo, Italy.*
[4] *Université Paris Cité and CNRS, ITODYS, F-75006, Paris France.*
* Email: luisa.danna@unipa.it

* Email: alessio.terenzi@unipa.it

* Email: uperricone@fondazionerimed.com

‡ L.D. and S.C. contributed equally to this work

Supplementary Information

In this section, a diverse range of results are presented for varying selections of features. The most effective models are highlighted in bold.

SPLIT 80:10:10

This subsection presents results obtained using a train-test split, where 80% of the data is used for training, 10% is used for validation, and the remaining 10% is used for testing.

Table S1. Performance of the Random Forest model with Mutual Information selection on different numbers of features.

Model	SS	Accuracy	Precision	Recall	F1	# features
RF	Mutual Info	0.7983	0.7727	0.7083	0.7391	50
RF	Mutual Info	0.7983	0.8000	0.6667	0.7273	60
RF	Mutual Info	0.8151	0.8095	0.7083	0.7556	70
RF	Mutual Info	0.8151	0.8095	0.7083	0.7556	80
RF	Mutual Info	0.8487	0.8571	0.7500	0.8000	90
RF	Mutual Info	0.8067	0.8049	0.6875	0.7416	100
RF	Mutual Info	0.7983	0.7857	0.6875	0.7333	110
RF	Mutual Info	0.8235	0.8293	0.7083	0.7640	120
RF	Mutual Info	0.8235	0.8293	0.7083	0.7640	130
RF	Mutual Info	0.8235	0.8140	0.7292	0.7692	140
RF	Mutual Info	0.7899	0.7949	0.6458	0.7126	150
RF	Mutual Info	0.7899	0.7805	0.6667	0.7191	160
RF	Mutual Info	0.7983	0.7857	0.6875	0.7333	170
RF	Mutual Info	0.8151	0.8250	0.6875	0.7500	180
RF	Mutual Info	0.7899	0.7805	0.6667	0.7191	190
RF	Mutual Info	0.7983	0.7857	0.6875	0.7333	200
RF	Mutual Info	0.7899	0.7805	0.6667	0.7191	210
RF	Mutual Info	0.7647	0.7500	0.6250	0.6818	220
RF	Mutual Info	0.8067	0.8049	0.6875	0.7416	230
RF	Mutual Info	0.8067	0.8205	0.6667	0.7356	240
RF	Mutual Info	0.8235	0.8293	0.7083	0.7640	250
**RF**	**Mutual Info**	**0.8319**	**0.8333**	**0.7292**	**0.7778**	**260**
RF	Mutual Info	0.8067	0.8205	0.6667	0.7356	270
RF	Mutual Info	0.8151	0.8250	0.6875	0.7500	280
RF	Mutual Info	0.8067	0.7907	0.7083	0.7473	290
RF	Mutual Info	0.8067	0.8049	0.6875	0.7416	300

Table S2. Performance of the Random Forest model with ANOVA selection on different numbers of features.

Model	SS	Accuracy	Precision	Recall	F1	# features
RF	ANOVA	0.7815	0.7895	0.6250	0.6977	50
**RF**	**ANOVA**	**0.8151**	**0.7955**	**0.7292**	**0.7609**	**60**
RF	ANOVA	0.7983	0.8000	0.6667	0.7273	70
RF	ANOVA	0.7983	0.7857	0.6875	0.7333	80
RF	ANOVA	0.7983	0.7857	0.6875	0.7333	90
RF	ANOVA	0.7731	0.7442	0.6667	0.7033	100
RF	ANOVA	0.8067	0.7907	0.7083	0.7473	110
RF	ANOVA	0.7647	0.7381	0.6458	0.6889	120
RF	ANOVA	0.7899	0.7674	0.6875	0.7253	130
RF	ANOVA	0.7815	0.7750	0.6458	0.7045	140
RF	ANOVA	0.7815	0.7619	0.6667	0.7111	150
RF	ANOVA	0.7731	0.7442	0.6667	0.7033	160
RF	ANOVA	0.7647	0.7632	0.6042	0.6744	170
RF	ANOVA	0.7647	0.7500	0.6250	0.6818	180
RF	ANOVA	0.7647	0.7381	0.6458	0.6889	190
RF	ANOVA	0.7899	0.7949	0.6458	0.7126	200
RF	ANOVA	0.7899	0.7674	0.6875	0.7253	210
RF	ANOVA	0.7647	0.7500	0.6250	0.6818	220
RF	ANOVA	0.7647	0.7500	0.6250	0.6818	230
RF	ANOVA	0.7647	0.7632	0.6042	0.6744	240
RF	ANOVA	0.7731	0.7561	0.6458	0.6966	250
RF	ANOVA	0.7563	0.7436	0.6042	0.6667	260
RF	ANOVA	0.7815	0.7895	0.6250	0.6977	270
RF	ANOVA	0.7815	0.7619	0.6667	0.7111	280
RF	ANOVA	0.7647	0.7381	0.6458	0.6889	290
RF	ANOVA	0.7647	0.7632	0.6042	0.6744	300

Table S3. Performance of the Random Forest model with Chi2 selection on different numbers of features.

Model	SS	Accuracy	Precision	Recall	F1	# features
RF	Chi2	0.7815	0.8056	0.6042	0.6905	50
RF	Chi2	0.7983	0.8529	0.6042	0.7073	60
**RF**	**Chi2**	**0.8319**	**0.8500**	**0.7083**	**0.7727**	**70**
RF	Chi2	0.8235	0.8293	0.7083	0.7640	80
RF	Chi2	0.7983	0.7857	0.6875	0.7333	90
RF	Chi2	0.8067	0.8378	0.6458	0.7294	100
RF	Chi2	0.7731	0.7692	0.6250	0.6897	110
RF	Chi2	0.8067	0.8049	0.6875	0.7416	120
RF	Chi2	0.7983	0.8000	0.6667	0.7273	130
RF	Chi2	0.7983	0.7857	0.6875	0.7333	140
RF	Chi2	0.7815	0.8056	0.6042	0.6905	150
RF	Chi2	0.7983	0.7857	0.6875	0.7333	160
RF	Chi2	0.8067	0.7907	0.7083	0.7473	170
RF	Chi2	0.7899	0.7805	0.6667	0.7191	180
RF	Chi2	0.8067	0.8049	0.6875	0.7416	190
RF	Chi2	0.8067	0.8205	0.6667	0.7356	200
RF	Chi2	0.7815	0.7619	0.6667	0.7111	210
RF	Chi2	0.8067	0.8205	0.6667	0.7356	220
RF	Chi2	0.7983	0.7857	0.6875	0.7333	230
RF	Chi2	0.7983	0.8000	0.6667	0.7273	240
RF	Chi2	0.7899	0.7805	0.6667	0.7191	250
RF	Chi2	0.7983	0.8000	0.6667	0.7273	260
RF	Chi2	0.8067	0.8205	0.6667	0.7356	270
RF	Chi2	0.7983	0.7857	0.6875	0.7333	280
RF	Chi2	0.7983	0.8000	0.6667	0.7273	290
RF	Chi2	0.8067	0.8049	0.6875	0.7416	300

Table S4: Performance of the Random Forest model with Random Forest Importance selection on different numbers of features.

Model	SS	Accuracy	Precision	Recall	F1	# features
RF	RFI	0.7815	0.7500	0.6875	0.7174	60
RF	RFI	0.7647	0.7381	0.6458	0.6889	70
RF	RFI	0.7983	0.7857	0.6875	0.7333	80
RF	RFI	0.7983	0.8000	0.6667	0.7273	90
RF	RFI	0.7983	0.7727	0.7083	0.7391	100
RF	RFI	0.8067	0.8049	0.6875	0.7416	110
RF	RFI	0.8067	0.7907	0.7083	0.7473	120
RF	RFI	0.7899	0.7805	0.6667	0.7191	130
RF	RFI	0.7899	0.7674	0.6875	0.7253	140
RF	RFI	0.7899	0.7805	0.6667	0.7191	150
RF	RFI	0.7899	0.7674	0.6875	0.7253	160
RF	RFI	0.8067	0.7907	0.7083	0.7473	170
RF	RFI	0.7899	0.7674	0.6875	0.7253	180
RF	RFI	0.7647	0.7381	0.6458	0.6889	190
RF	RFI	0.8067	0.8049	0.6875	0.7416	200
RF	RFI	0.7899	0.7805	0.6667	0.7191	210
RF	RFI	0.7647	0.7500	0.6250	0.6818	220
RF	RFI	0.7983	0.7727	0.7083	0.7391	230
RF	RFI	0.7815	0.7750	0.6458	0.7045	240
RF	RFI	0.8067	0.7907	0.7083	0.7473	250
**RF**	**RFI**	**0.8151**	**0.8250**	**0.6875**	**0.7500**	**260**
RF	RFI	0.7983	0.7857	0.6875	0.7333	270
RF	RFI	0.8067	0.8049	0.6875	0.7416	280
RF	RFI	0.7815	0.7895	0.6250	0.6977	290
RF	RFI	0.8067	0.8049	0.6875	0.7416	300

Table S5. Performance of the Decision Tree model with Mutual Information selection on different numbers of features.

Model	SS	Accuracy	Precision	Recall	F1-score	# features
DT	Mutual Info	0.7479	0.6800	0.7083	0.6939	50
DT	Mutual Info	0.7227	0.6471	0.6875	0.6667	60
DT	Mutual Info	0.7311	0.6600	0.6875	0.6735	70
DT	Mutual Info	0.7395	0.6889	0.6458	0.6667	80
DT	Mutual Info	0.7731	0.7561	0.6458	0.6966	90
DT	Mutual Info	0.7563	0.7209	0.6458	0.6813	100
DT	Mutual Info	0.7647	0.7273	0.6667	0.6957	110
DT	Mutual Info	0.7731	0.7333	0.6875	0.7097	120
DT	Mutual Info	0.7563	0.7209	0.6458	0.6813	130
**DT**	**Mutual Info**	**0.8067**	**0.7551**	**0.7708**	**0.7629**	**140**
DT	Mutual Info	0.7563	0.7021	0.6875	0.6947	150
DT	Mutual Info	0.7731	0.7333	0.6875	0.7097	160
DT	Mutual Info	0.7563	0.6863	0.7292	0.7071	170
DT	Mutual Info	0.7479	0.6957	0.6667	0.6809	180
DT	Mutual Info	0.7647	0.7000	0.7292	0.7143	190
DT	Mutual Info	0.7143	0.6296	0.7083	0.6667	200
DT	Mutual Info	0.7395	0.6977	0.6250	0.6593	210
DT	Mutual Info	0.7479	0.7045	0.6458	0.6739	220
DT	Mutual Info	0.7563	0.7021	0.6875	0.6947	230
DT	Mutual Info	0.7479	0.6875	0.6875	0.6875	240
DT	Mutual Info	0.7647	0.7174	0.6875	0.7021	250
DT	Mutual Info	0.7731	0.7143	0.7292	0.7216	260
DT	Mutual Info	0.7479	0.6800	0.7083	0.6939	270
DT	Mutual Info	0.7647	0.7273	0.6667	0.6957	280
DT	Mutual Info	0.7563	0.7317	0.6250	0.6742	290
DT	Mutual Info	0.7479	0.6957	0.6667	0.6809	300

Table S6. Performance of the Decision Tree model with ANOVA selection on different numbers of features.

Model	SS	Accuracy	Precision	Recall	F1-score	n features
DT	ANOVA	0.7394	0.6666	0.7083	0.6868	50
DT	ANOVA	0.7563	0.6862	0.7291	0.7070	60
DT	ANOVA	0.7647	0.7173	0.6875	0.7021	70
DT	ANOVA	0.7647	0.7173	0.6875	0.7021	80
DT	ANOVA	0.7647	0.6851	0.7708	0.7254	90
DT	ANOVA	0.7815	0.7500	0.6875	0.7173	100
DT	ANOVA	0.7226	0.6595	0.6458	0.6526	110
DT	ANOVA	0.7563	0.7111	0.6666	0.6881	120
DT	ANOVA	0.7731	0.7333	0.6875	0.7096	130
DT	ANOVA	0.7899	0.7346	0.7500	0.7422	140
**DT**	**ANOVA**	**0.7983**	**0.7608**	**0.7291**	**0.7446**	**150**
DT	ANOVA	0.7731	0.7234	0.7083	0.7157	160
DT	ANOVA	0.7899	0.7173	0.6875	0.7021	170
DT	ANOVA	0.7983	0.7446	0.7291	0.7368	180
DT	ANOVA	0.7731	0.7234	0.7083	0.7157	190
DT	ANOVA	0.7563	0.7111	0.6666	0.6881	200
DT	ANOVA	0.6974	0.6363	0.5833	0.6086	210
DT	ANOVA	0.7058	0.6444	0.6041	0.6236	220
DT	ANOVA	0.7563	0.7021	0.6875	0.6947	230
DT	ANOVA	0.6974	0.7045	0.6458	0.6739	240
DT	ANOVA	0.6806	0.6041	0.6041	0.6041	250
DT	ANOVA	0.6806	0.6041	0.6041	0.6041	260
DT	ANOVA	0.7310	0.6818	0.6250	0.6521	270
DT	ANOVA	0.7226	0.6829	0.5833	0.6292	280
DT	ANOVA	0.7310	0.6904	0.6041	0.6444	290
DT	ANOVA	0.7226	0.6666	0.6250	0.6451	300

Table S7. Performance of the Decision Tree model with Chi2 selection on different numbers of features.

Model	SS	Accuracy	Precision	Recall	F1	# features
DT	Chi2	0.7563	0.7436	0.6042	0.6667	50
DT	Chi2	0.7899	0.7805	0.6667	0.7191	60
DT	Chi2	0.7311	0.6600	0.6875	0.6735	70
DT	Chi2	0.7647	0.7083	0.7083	0.7083	80
DT	Chi2	0.7311	0.6600	0.6875	0.6735	90
DT	Chi2	0.6639	0.5769	0.6250	0.6000	100
DT	Chi2	0.6975	0.6071	0.7083	0.6538	110
DT	Chi2	0.7395	0.6735	0.6875	0.6804	120
DT	Chi2	0.7227	0.6531	0.6667	0.6598	130
DT	Chi2	0.7479	0.6731	0.7292	0.7000	140
DT	Chi2	0.7563	0.6862	0.7292	0.7070	150
DT	Chi2	0.7478	0.6956	0.6666	0.6808	160
DT	Chi2	0.7142	0.6346	0.6875	0.6600	170
**DT**	**Chi2**	**0.7899**	**0.7555**	**0.7083**	**0.7311**	**180**
DT	Chi2	0.7394	0.6734	0.6875	0.6804	190
DT	Chi2	0.7815	0.7391	0.7083	0.7234	200
DT	Chi2	0.7563	0.6862	0.7291	0.7070	210
DT	Chi2	0.7394	0.6888	0.6458	0.6666	220
DT	Chi2	0.7394	0.6888	0.6458	0.6666	230
DT	Chi2	0.7310	0.6739	0.6458	0.6595	240
DT	Chi2	0.7478	0.6800	0.7083	0.6938	250
DT	Chi2	0.7647	0.6923	0.7500	0.7200	260
DT	Chi2	0.6974	0.6250	0.6250	0.6250	270
DT	Chi2	0.7142	0.6400	0.6666	0.6530	280
DT	Chi2	0.7142	0.6296	0.7083	0.6666	290
DT	Chi2	0.7226	0.6595	0.6458	0.6526	300

Table S8. Performance of the Naive Bayes model with ANOVA selection on different numbers of features.

**Model**	**SS**	**Accuracy**	**Precision**	**Recall**	**F1**	**# features**
NB	ANOVA	0.6387	0.5510	0.5625	0.5567	60
NB	ANOVA	0.6134	0.5200	0.5417	0.5306	70
NB	ANOVA	0.6218	0.5306	0.5417	0.5361	80
NB	ANOVA	0.6303	0.5400	0.5625	0.5510	90
NB	ANOVA	0.6387	0.5510	0.5625	0.5567	100
NB	ANOVA	0.6303	0.5417	0.5417	0.5417	110
NB	ANOVA	0.6387	0.5532	0.5417	0.5474	120
NB	ANOVA	0.6303	0.5400	0.5625	0.5510	130
NB	ANOVA	0.6387	0.5510	0.5625	0.5567	140
NB	ANOVA	0.6303	0.5400	0.5625	0.5510	150
NB	ANOVA	0.6218	0.5294	0.5625	0.5455	160
NB	ANOVA	0.6218	0.5294	0.5625	0.5455	170
NB	ANOVA	0.6303	0.5385	0.5833	0.5600	180
**NB**	**ANOVA**	**0.6387**	**0.5490**	**0.5833**	**0.5657**	**190**
**NB**	**ANOVA**	**0.6387**	**0.5490**	**0.5833**	**0.5657**	**200**
NB	ANOVA	0.6303	0.5400	0.5625	0.5510	210
NB	ANOVA	0.6387	0.5510	0.5625	0.5567	220
NB	ANOVA	0.6387	0.5510	0.5625	0.5567	230
NB	ANOVA	0.6303	0.5400	0.5625	0.5510	240
NB	ANOVA	0.6471	0.5625	0.5625	0.5625	250
NB	ANOVA	0.6387	0.5510	0.5625	0.5567	260
NB	ANOVA	0.6303	0.5417	0.5417	0.5417	270
NB	ANOVA	0.6303	0.5400	0.5625	0.5510	280
NB	ANOVA	0.6303	0.5400	0.5625	0.5510	290
NB	ANOVA	0.6387	0.5510	0.5625	0.5567	300

Table S9. Performance of the Naive Bayes model with Chi2 selection on different numbers of features.

Model	SS	Accuracy	Precision	Recall	F1	# features
NB	Chi2	0.6975	0.7000	0.4375	0.5385	60
NB	Chi2	0.6975	0.6875	0.4583	0.5500	70
NB	Chi2	0.6723	0.6216	0.4792	0.5412	80
NB	Chi2	0.6807	0.6389	0.4792	0.5476	90
NB	Chi2	0.6639	0.6053	0.4792	0.5349	100
NB	Chi2	0.6807	0.6316	0.5000	0.5581	110
NB	Chi2	0.6891	0.6410	0.5208	0.5747	120
NB	Chi2	0.7059	0.6667	0.5417	0.5977	130
NB	Chi2	0.6723	0.5763	0.7083	0.6355	140
NB	Chi2	0.6555	0.5507	0.7917	0.6496	150
NB	Chi2	0.6555	0.5507	0.7917	0.6496	160
NB	Chi2	0.6723	0.5672	0.7917	0.6609	170
NB	Chi2	0.6387	0.5342	0.8125	0.6446	180
NB	Chi2	0.6723	0.5672	0.7917	0.6609	190
NB	Chi2	0.6807	0.5758	0.7917	0.6667	200
**NB**	**Chi2**	**0.6891**	**0.5846**	**0.7917**	**0.6726**	**210**
NB	Chi2	0.6723	0.5714	0.7500	0.6486	220
NB	Chi2	0.6639	0.5645	0.7292	0.6364	230
NB	Chi2	0.6723	0.5672	0.7917	0.6609	240
NB	Chi2	0.6387	0.5352	0.7917	0.6387	250
NB	Chi2	0.6303	0.5286	0.7708	0.6271	260
NB	Chi2	0.6387	0.5362	0.7708	0.6325	270
NB	Chi2	0.6975	0.6200	0.6458	0.6327	280
NB	Chi2	0.6975	0.6200	0.6458	0.6327	290
NB	Chi2	0.6975	0.6200	0.6458	0.6327	300

Table S10. Performance of the Naive Bayes model with Mutual Info selection on different numbers of features.

**Model**	**Selection Method**	**Accuracy**	**Precision**	**Recall**	**F1-score**	**n features**
NB	Mutual Info	0.6555	0.5660	0.6250	0.5941	60
NB	Mutual Info	0.6723	0.5957	0.5833	0.5895	70
NB	Mutual Info	0.6471	0.5556	0.6250	0.5882	80
NB	Mutual Info	0.6723	0.5818	0.6667	0.6214	90
**NB**	**Mutual Info**	**0.6891**	**0.6000**	**0.6875**	**0.6408**	**100**
NB	Mutual Info	0.6639	0.5741	0.6458	0.6078	110
NB	Mutual Info	0.6471	0.5536	0.6458	0.5962	120
NB	Mutual Info	0.6134	0.5161	0.6667	0.5818	130
NB	Mutual Info	0.6218	0.5263	0.6250	0.5714	140
NB	Mutual Info	0.6303	0.5357	0.6250	0.5769	150
NB	Mutual Info	0.6387	0.5424	0.6667	0.5981	160
NB	Mutual Info	0.6387	0.5439	0.6458	0.5905	170
NB	Mutual Info	0.6387	0.5455	0.6250	0.5825	180
NB	Mutual Info	0.6387	0.5472	0.6042	0.5743	190
NB	Mutual Info	0.6387	0.5490	0.5833	0.5657	200
NB	Mutual Info	0.6387	0.5439	0.6458	0.5905	210
NB	Mutual Info	0.6303	0.5370	0.6042	0.5686	220
NB	Mutual Info	0.6639	0.5741	0.6458	0.6078	230
NB	Mutual Info	0.6471	0.5536	0.6458	0.5962	240
NB	Mutual Info	0.6555	0.5686	0.6042	0.5859	250
NB	Mutual Info	0.6723	0.5882	0.6250	0.6061	260
NB	Mutual Info	0.6471	0.5600	0.5833	0.5714	270
NB	Mutual Info	0.6471	0.5714	0.5000	0.5333	280
NB	Mutual Info	0.6471	0.5625	0.5625	0.5625	290
NB	Mutual Info	0.6050	0.5000	0.5417	0.5253	300

Summary of Feature Selection using CROSS VALIDATION

F1-Score by Model, Feature Selection Method, and Number of Features

Table S11. To evaluate model performance, we focus on the **F1-score**, as it best captures the balance between precision and recall. For each combination of model, feature selection method, and number of selected features, we report the **mean F1-score** across cross-validation folds. This allows a fair comparison of model effectiveness under different feature selection strategies and feature set sizes.

Model	SS	# features	Accuracy	Precision	Recall	F1
DecisionTree	ANOVA	50	0.78616	0.7377	0.7272	0.7307
DecisionTree	ANOVA	60	0.7769	0.7245	0.7203	0.7191
DecisionTree	ANOVA	70	0.7760	0.7200	0.7209	0.7175
DecisionTree	ANOVA	80	0.7727	0.7082	0.7328	0.7186
DecisionTree	ANOVA	90	0.7735	0.7151	0.7230	0.7160
DecisionTree	ANOVA	100	0.7744	0.7230	0.7185	0.7162
DecisionTree	ANOVA	110	0.7483	0.6892	0.6990	0.6878
DecisionTree	ANOVA	120	0.7558	0.6959	0.6921	0.6907
DecisionTree	ANOVA	130	0.7491	0.6907	0.6863	0.6843
DecisionTree	ANOVA	140	0.7642	0.7026	0.7002	0.6998
DecisionTree	ANOVA	150	0.7777	0.7145	0.7321	0.7216
DecisionTree	ANOVA	160	0.7802	0.7106	0.7472	0.7271
DecisionTree	ANOVA	170	0.7626	0.6972	0.7229	0.7075
DecisionTree	ANOVA	180	0.7592	0.6890	0.7262	0.7047
DecisionTree	ANOVA	190	0.7802	0.7107	0.7585	0.7323
DecisionTree	ANOVA	200	0.7735	0.7113	0.7393	0.7217
DecisionTree	ANOVA	210	0.7726	0.7081	0.7354	0.7192
DecisionTree	ANOVA	220	0.7962	0.7368	0.7646	0.7486
DecisionTree	ANOVA	230	0.7853	0.7290	0.7388	0.7322
DecisionTree	ANOVA	240	0.7937	0.7530	0.7238	0.7352
DecisionTree	ANOVA	250	0.7878	0.7346	0.7379	0.7336
DecisionTree	ANOVA	260	0.7735	0.7223	0.7163	0.7154
DecisionTree	ANOVA	270	0.7768	0.7194	0.7231	0.7192
DecisionTree	ANOVA	280	0.7769	0.7244	0.7267	0.7206
DecisionTree	ANOVA	290	0.7701	0.7189	0.7075	0.7096
DecisionTree	ANOVA	300	0.7659	0.7111	0.7159	0.7090
DecisionTree	Chi2	50	0.7718	0.7088	0.7332	0.7177
DecisionTree	Chi2	60	0.7811	0.7242	0.7388	0.7274
DecisionTree	Chi2	70	0.7676	0.6977	0.7411	0.7165
DecisionTree	Chi2	80	0.7718	0.7095	0.7304	0.7175
DecisionTree	Chi2	90	0.7760	0.7278	0.7245	0.7194

DecisionTree	Chi2	100	0.7643	0.7040	0.7088	0.7044
DecisionTree	Chi2	110	0.7777	0.7182	0.7268	0.7204
DecisionTree	Chi2	120	0.7837	0.7251	0.7471	0.7332
DecisionTree	Chi2	130	0.7861	0.7216	0.7563	0.7368
DecisionTree	Chi2	140	0.7793	0.7202	0.7336	0.7246
DecisionTree	Chi2	150	0.7642	0.6995	0.7157	0.7058
DecisionTree	Chi2	160	0.7743	0.7088	0.7416	0.7218
DecisionTree	Chi2	170	0.7785	0.7117	0.7506	0.7272
DecisionTree	Chi2	180	0.7769	0.7107	0.7449	0.7257
DecisionTree	Chi2	190	0.7668	0.6997	0.7215	0.7086
DecisionTree	Chi2	200	0.7777	0.7162	0.7353	0.7228
DecisionTree	Chi2	210	0.7626	0.6947	0.7264	0.7079
DecisionTree	Chi2	220	0.7584	0.6921	0.7082	0.6986
DecisionTree	Chi2	230	0.7542	0.6834	0.7186	0.6986
DecisionTree	Chi2	240	0.7660	0.7065	0.7122	0.7064
DecisionTree	Chi2	250	0.7559	0.6833	0.7258	0.7013
DecisionTree	Chi2	260	0.7642	0.6997	0.7234	0.7092
DecisionTree	Chi2	270	0.7634	0.6998	0.7245	0.7086
DecisionTree	Chi2	280	0.7710	0.7089	0.7340	0.7188
DecisionTree	Chi2	290	0.7727	0.7084	0.7391	0.7192
DecisionTree	Chi2	300	0.7592	0.6859	0.7355	0.7071
DecisionTree	MutualInfo	50	0.7929	0.7489	0.7339	0.7383
DecisionTree	MutualInfo	60	0.7879	0.7373	0.7384	0.7346
DecisionTree	MutualInfo	70	0.7920	0.7360	0.74939	0.7409
DecisionTree	MutualInfo	80	0.79463	0.7456	0.7450	0.7427
DecisionTree	MutualInfo	90	0.7988	0.7504	0.7420	0.7446
DecisionTree	MutualInfo	100	0.7903	0.7404	0.7333	0.7340
DecisionTree	MutualInfo	110	0.7786	0.7190	0.7390	0.7270
DecisionTree	MutualInfo	120	0.7971	0.7529	0.7428	0.7453
DecisionTree	MutualInfo	130	0.7938	0.7317	0.7624	0.7461
DecisionTree	MutualInfo	140	0.7895	0.7362	0.7423	0.7372
DecisionTree	MutualInfo	150	0.8005	0.7459	0.7634	0.7527
DecisionTree	MutualInfo	160	0.7752	0.7087	0.7479	0.7258
DecisionTree	MutualInfo	170	0.7920	0.7331	0.7598	0.7445
DecisionTree	MutualInfo	180	0.7853	0.7212	0.7519	0.7350
DecisionTree	MutualInfo	190	0.7837	0.7195	0.7606	0.7383
DecisionTree	MutualInfo	200	0.7996	0.7394	0.7727	0.7544
DecisionTree	MutualInfo	210	0.7828	0.7240	0.7431	0.7314
DecisionTree	MutualInfo	220	0.7811	0.7179	0.7532	0.7332
DecisionTree	MutualInfo	230	0.7677	0.6983	0.7380	0.7157
DecisionTree	MutualInfo	240	0.7634	0.6968	0.7213	0.7076

DecisionTree	MutualInfo	250	0.7710	0.7068	0.7461	0.7222
DecisionTree	MutualInfo	260	0.7903	0.7272	0.7571	0.7405
DecisionTree	MutualInfo	270	0.7769	0.7087	0.7573	0.7305
DecisionTree	MutualInfo	280	0.7719	0.7160	0.7169	0.7146
DecisionTree	MutualInfo	290	0.7592	0.6921	0.7286	0.7067
DecisionTree	MutualInfo	300	0.7701	0.6974	0.7376	0.7163

Model	SS	Num - Features	Accuracy	Precision	Recall	F1
NaiveBayes	ANOVA	50	0.6842	0.6063	0.6241	0.6091
NaiveBayes	ANOVA	60	0.6893	0.6098	0.6416	0.6202
NaiveBayes	ANOVA	70	0.6842	0.6020	0.6416	0.6163
NaiveBayes	ANOVA	80	0.6868	0.6055	0.6404	0.6173
NaiveBayes	ANOVA	90	0.6809	0.5983	0.6407	0.6129
NaiveBayes	ANOVA	100	0.6809	0.5987	0.6427	0.6143
NaiveBayes	ANOVA	110	0.6809	0.6012	0.6270	0.6084
NaiveBayes	ANOVA	120	0.6842	0.6036	0.6384	0.6150
NaiveBayes	ANOVA	130	0.6851	0.6052	0.6362	0.6148
NaiveBayes	ANOVA	140	0.6885	0.6069	0.6458	0.6210
NaiveBayes	ANOVA	150	0.6918	0.6111	0.6460	0.6230
NaiveBayes	ANOVA	160	0.6851	0.6021	0.6414	0.6164
NaiveBayes	ANOVA	170	0.6851	0.6010	0.6463	0.6176
NaiveBayes	ANOVA	180	0.6851	0.6019	0.6422	0.6160
NaiveBayes	ANOVA	190	0.6876	0.6063	0.6447	0.6197
NaiveBayes	ANOVA	200	0.6868	0.6049	0.6449	0.6186
NaiveBayes	ANOVA	210	0.6876	0.6056	0.6446	0.6195
NaiveBayes	ANOVA	220	0.6860	0.6038	0.6446	0.6183
NaiveBayes	ANOVA	230	0.6851	0.6046	0.6361	0.6144
NaiveBayes	ANOVA	240	0.6877	0.6072	0.6383	0.6168
NaiveBayes	ANOVA	250	0.6902	0.6110	0.6385	0.6189
NaiveBayes	ANOVA	260	0.6876	0.6077	0.6342	0.6149
NaiveBayes	ANOVA	270	0.6885	0.6098	0.6299	0.6142
NaiveBayes	ANOVA	280	0.6851	0.6059	0.6184	0.6067
NaiveBayes	ANOVA	290	0.6834	0.6056	0.6122	0.6038
NaiveBayes	ANOVA	300	0.6826	0.6029	0.6160	0.6044
NaiveBayes	Chi2	50	0.6818	0.6588	0.4245	0.5110
NaiveBayes	Chi2	60	0.6784	0.6541	0.4488	0.5223
NaiveBayes	Chi2	70	0.6910	0.6582	0.4985	0.5583
NaiveBayes	Chi2	80	0.6893	0.6483	0.5050	0.5518
NaiveBayes	Chi2	90	0.6910	0.6363	0.5265	0.5661
NaiveBayes	Chi2	100	0.6969	0.6422	0.5560	0.5906
NaiveBayes	Chi2	110	0.7020	0.6504	0.5805	0.6001
NaiveBayes	Chi2	120	0.7003	0.6304	0.6252	0.6164
NaiveBayes	Chi2	130	0.6809	0.6027	0.6576	0.6129
NaiveBayes	Chi2	140	0.6650	0.5825	0.6742	0.6110
NaiveBayes	Chi2	150	0.6624	0.5757	0.6836	0.6130

NaiveBayes	Chi2	160	0.6583	0.5651	0.7299	0.6250
NaiveBayes	Chi2	170	0.6575	0.5644	0.7469	0.6322
NaiveBayes	Chi2	180	0.6608	0.5661	0.7525	0.6364
NaiveBayes	Chi2	190	0.6499	0.5489	0.7565	0.6302
NaiveBayes	Chi2	200	0.6490	0.5477	0.7754	0.6368
NaiveBayes	Chi2	210	0.6440	0.5400	0.7980	0.6390
NaiveBayes	Chi2	220	0.6330	0.5353	0.8050	0.6355
NaiveBayes	Chi2	230	0.6297	0.5288	0.8210	0.6383
NaiveBayes	Chi2	240	0.6330	0.5327	0.8228	0.6407
NaiveBayes	Chi2	250	0.6330	0.5328	0.8204	0.6404
NaiveBayes	Chi2	260	0.6313	0.5310	0.8121	0.6363
NaiveBayes	Chi2	270	0.6423	0.5398	0.8062	0.6417
NaiveBayes	Chi2	280	0.6153	0.5226	0.8372	0.6343
NaiveBayes	Chi2	290	0.6170	0.5199	0.8572	0.6420
NaiveBayes	Chi2	300	0.6144	0.5166	0.8581	0.6400
NaiveBayes	MutualInfo	50	0.6809	0.5945	0.6504	0.6162
NaiveBayes	MutualInfo	60	0.6725	0.5868	0.6283	0.6019
NaiveBayes	MutualInfo	70	0.6817	0.6001	0.6378	0.6123
NaiveBayes	MutualInfo	80	0.6759	0.5956	0.6145	0.5990
NaiveBayes	MutualInfo	90	0.6759	0.6024	0.5830	0.5849
NaiveBayes	MutualInfo	100	0.6817	0.6010	0.6040	0.5995
NaiveBayes	MutualInfo	110	0.6834	0.6058	0.6110	0.6026
NaiveBayes	MutualInfo	120	0.6834	0.6036	0.6046	0.6002
NaiveBayes	MutualInfo	130	0.6792	0.5985	0.6468	0.6131
NaiveBayes	MutualInfo	140	0.6759	0.5908	0.6528	0.6135
NaiveBayes	MutualInfo	150	0.6759	0.5931	0.6350	0.6063
NaiveBayes	MutualInfo	160	0.6826	0.5984	0.6549	0.6196
NaiveBayes	MutualInfo	170	0.6750	0.5905	0.6470	0.6104
NaiveBayes	MutualInfo	180	0.6759	0.5912	0.6382	0.6072
NaiveBayes	MutualInfo	190	0.6758	0.5919	0.6353	0.6063
NaiveBayes	MutualInfo	200	0.6759	0.5947	0.6275	0.6040
NaiveBayes	MutualInfo	210	0.6725	0.5918	0.6192	0.5981
NaiveBayes	MutualInfo	220	0.6759	0.5929	0.6297	0.6050
NaiveBayes	MutualInfo	230	0.6750	0.5921	0.6155	0.5975
NaiveBayes	MutualInfo	240	0.6742	0.5904	0.6184	0.5990
NaiveBayes	MutualInfo	250	0.6708	0.5883	0.6091	0.5930
NaiveBayes	MutualInfo	260	0.6767	0.5970	0.6072	0.5964
NaiveBayes	MutualInfo	270	0.6809	0.6007	0.6165	0.6037
NaiveBayes	MutualInfo	280	0.6750	0.5939	0.6155	0.5994
NaiveBayes	MutualInfo	290	0.6750	0.5954	0.6123	0.5972
NaiveBayes	MutualInfo	300	0.6784	0.5976	0.6171	0.6024

Model	SS	Num - Features	Accuracy	Precision	Recall	F1
RandomForest	ANOVA	50	0.8459	0.8212	0.7849	0.8007
RandomForest	ANOVA	60	0.8442	0.8142	0.7950	0.8013
RandomForest	ANOVA	70	0.8467	0.8216	0.7913	0.8036
RandomForest	ANOVA	80	0.8509	0.8255	0.7970	0.8087
RandomForest	ANOVA	90	0.8518	0.8252	0.7979	0.8097
RandomForest	ANOVA	100	0.8434	0.8196	0.7804	0.7974
RandomForest	ANOVA	110	0.8442	0.8116	0.7978	0.8018
RandomForest	ANOVA	120	0.8451	0.8199	0.7865	0.8002
RandomForest	ANOVA	130	0.8509	0.8261	0.7977	0.8093
RandomForest	ANOVA	140	0.8510	0.8248	0.7999	0.8089
RandomForest	ANOVA	150	0.8543	0.8309	0.8011	0.8130
RandomForest	ANOVA	160	0.8484	0.8233	0.7893	0.8040
RandomForest	ANOVA	170	0.8568	0.8321	0.8069	0.8168
RandomForest	ANOVA	180	0.8518	0.8325	0.7914	0.8083
RandomForest	ANOVA	190	0.8451	0.8185	0.78621	0.8003
RandomForest	ANOVA	200	0.8468	0.8248	0.7844	0.8019
RandomForest	ANOVA	210	0.8459	0.8184	0.7896	0.8013
RandomForest	ANOVA	220	0.8551	0.8365	0.7951	0.8128
RandomForest	ANOVA	230	0.8484	0.8198	0.7967	0.8058
RandomForest	ANOVA	240	0.8417	0.8216	0.7700	0.7926
RandomForest	ANOVA	250	0.8468	0.8289	0.7779	0.8001
RandomForest	ANOVA	260	0.8560	0.8353	0.8004	0.8148

RandomForest	ANOVA	270	0.8535	0.8310	0.7968	0.8114
RandomForest	ANOVA	280	0.8467	0.8224	0.7886	0.8024
RandomForest	ANOVA	290	0.8585	0.8473	0.7907	0.8150
RandomForest	ANOVA	300	0.8467	0.8257	0.7832	0.8017
RandomForest	Chi2	50	0.8602	0.8420	0.8013	0.8193
RandomForest	Chi2	60	0.8610	0.8399	0.8043	0.8203
RandomForest	Chi2	70	0.8501	0.8316	0.7882	0.8070
RandomForest	Chi2	80	0.8560	0.8371	0.7996	0.8148
RandomForest	Chi2	90	0.8543	0.8318	0.7993	0.8132
RandomForest	Chi2	100	0.8551	0.8342	0.80138	0.8149
RandomForest	Chi2	110	0.8585	0.8393	0.8030	0.8182
RandomForest	Chi2	120	0.8518	0.8243	0.8019	0.8106
RandomForest	Chi2	130	0.8577	0.8355	0.8034	0.8174
RandomForest	Chi2	140	0.8560	0.8390	0.7941	0.8137
RandomForest	Chi2	150	0.8543	0.8326	0.7996	0.8131
RandomForest	Chi2	160	0.8476	0.8257	0.7841	0.8020
RandomForest	Chi2	170	0.8534	0.8353	0.7901	0.8097
RandomForest	Chi2	180	0.8493	0.8263	0.7905	0.8054
RandomForest	Chi2	190	0.8526	0.8360	0.7871	0.8091
RandomForest	Chi2	200	0.8535	0.8268	0.8043	0.8129
RandomForest	Chi2	210	0.8568	0.8367	0.8035	0.8166
RandomForest	Chi2	220	0.8526	0.8308	0.7959	0.8107
RandomForest	Chi2	230	0.8551	0.8349	0.7973	0.8133
RandomForest	Chi2	240	0.8543	0.8338	0.7950	0.8119
RandomForest	Chi2	250	0.8450	0.8254	0.7829	0.8000

RandomForest	Chi2	260	0.8535	0.8368	0.7892	0.8099
RandomForest	Chi2	270	0.8560	0.8417	0.7893	0.8123
RandomForest	Chi2	280	0.8568	0.8375	0.7972	0.8146
RandomForest	Chi2	290	0.8560	0.8374	0.7989	0.8144
RandomForest	Chi2	300	0.8543	0.8384	0.7916	0.8112
RandomForest	MutualInfo	50	0.8484	0.8258	0.7894	0.8044
RandomForest	MutualInfo	60	0.8459	0.8219	0.7903	0.8025
RandomForest	MutualInfo	70	0.8467	0.8199	0.7928	0.8034
RandomForest	MutualInfo	80	0.8501	0.8345	0.78476	0.8055
RandomForest	MutualInfo	90	0.8577	0.8405	0.7987	0.8164
RandomForest	MutualInfo	100	0.8568	0.8370	0.8017	0.8164
RandomForest	MutualInfo	110	0.8534	0.8359	0.7933	0.8109
RandomForest	MutualInfo	120	0.8526	0.8390	0.7845	0.8084
RandomForest	MutualInfo	130	0.8510	0.8298	0.7938	0.8085
RandomForest	MutualInfo	140	0.8526	0.8361	0.7918	0.8101
RandomForest	MutualInfo	150	0.8585	0.8439	0.7943	0.8158
RandomForest	MutualInfo	160	0.8518	0.8312	0.7899	0.8079
RandomForest	MutualInfo	170	0.8509	0.8330	0.7887	0.8074
RandomForest	MutualInfo	180	0.8552	0.8375	0.7963	0.8131
RandomForest	MutualInfo	190	0.8577	0.8358	0.8025	0.8165
RandomForest	MutualInfo	200	0.8510	0.8276	0.7939	0.8079
RandomForest	MutualInfo	210	0.8509	0.8269	0.7949	0.8082
RandomForest	MutualInfo	220	0.8535	0.8351	0.7894	0.8095
RandomForest	MutualInfo	230	0.8585	0.8520	0.7840	0.8138
RandomForest	MutualInfo	240	0.8476	0.8267	0.7877	0.80423

RandomForest	MutualInfo	250	0.8518	0.8263	0.7993	0.8100
RandomForest	MutualInfo	260	0.8552	0.8337	0.7961	0.8126
RandomForest	MutualInfo	270	0.8611	0.8431	0.8060	0.8213
RandomForest	MutualInfo	280	0.8560	0.8390	0.7962	0.8145
RandomForest	MutualInfo	290	0.8619	0.8350	0.8156	0.8234
RandomForest	MutualInfo	300	0.8636	0.8431	0.8081	0.8237
RandomForest	RFI	50	0.8585	0.8324	0.8129	0.8198
RandomForest	RFI	60	0.8611	0.8396	0.8079	0.8213
RandomForest	RFI	70	0.8560	0.8308	0.8057	0.8152
RandomForest	RFI	80	0.8552	0.8346	0.7978	0.8135
RandomForest	RFI	90	0.8468	0.8223	0.7888	0.8023
RandomForest	RFI	100	0.8636	0.8470	0.8054	0.8235
RandomForest	RFI	110	0.8568	0.8303	0.8064	0.8163
RandomForest	RFI	120	0.8627	0.8484	0.8012	0.8217
RandomForest	RFI	130	0.8602	0.8461	0.7940	0.8172
RandomForest	RFI	140	0.8619	0.8475	0.8024	0.8211
RandomForest	RFI	150	0.8518	0.8347	0.7893	0.8078
RandomForest	RFI	160	0.8577	0.8420	0.7949	0.8154
RandomForest	RFI	170	0.8543	0.8379	0.7902	0.8103
RandomForest	RFI	180	0.8535	0.8397	0.7835	0.8086
RandomForest	RFI	190	0.8543	0.8344	0.7943	0.8114
RandomForest	RFI	200	0.8594	0.8416	0.7987	0.8172
RandomForest	RFI	210	0.8577	0.8461	0.7884	0.8132
RandomForest	RFI	220	0.8476	0.8252	0.7856	0.8025
RandomForest	RFI	230	0.8535	0.8341	0.7960	0.8112

RandomForest	RFI	240	0.8459	0.8340	0.7737	0.7985
RandomForest	RFI	250	0.8501	0.8292	0.7880	0.8060
RandomForest	RFI	260	0.8509	0.8267	0.7967	0.8083
RandomForest	RFI	270	0.8543	0.8357	0.7959	0.8118
RandomForest	RFI	280	0.8509	0.8348	0.7842	0.8060
RandomForest	RFI	290	0.8527	0.8431	0.7789	0.8067
RandomForest	RFI	300	0.8467	0.8235	0.7859	0.8017

Table S12. The test dataset comprises 27 molecules from our in-house database, used to validate the best-performing algorithm. N.a. = reference not available. All compounds have been synthesized and characterized but are not yet published. ΔT values represent the average of experimental measurements obtained from different G4 sequences. In green, the class assignment corresponds to the experimental findings.

Structure	Label	Experimental ΔT (˚C)*	Class assignment	Ref.
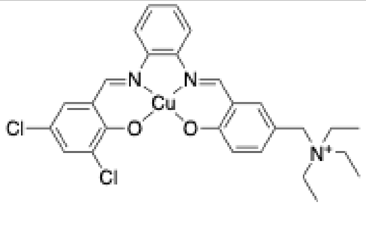	Cpd1	20.44	ACTIVE	n.a.
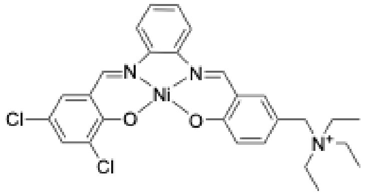	Cpd2	22.02	ACTIVE	n.a.
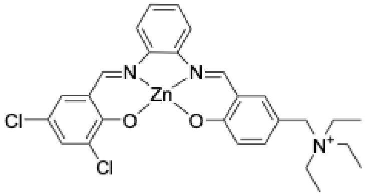	Cpd3	1.68	INACTIVE	1
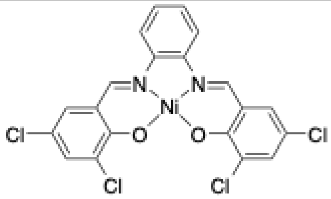	Cpd4	6.91	INACTIVE	2
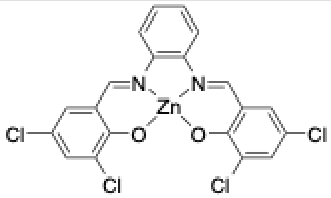	Cpd5	1.95	INACTIVE	2

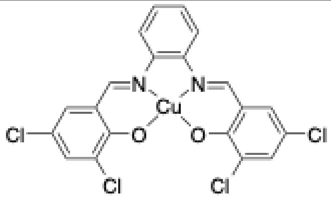	Cpd6	5.90	INACTIVE	2
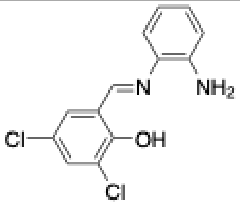	Cpd7	0.52	INACTIVE	1
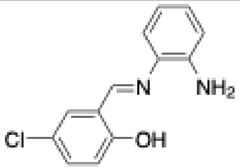	Cpd8	-0.18	INACTIVE	n.a.
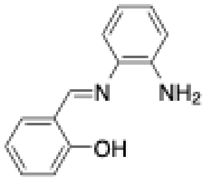	Cpd9	0.35	INACTIVE	n.a.
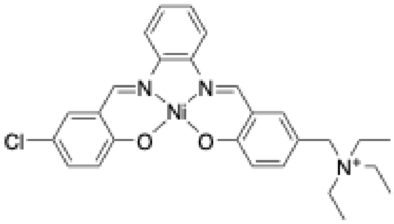	Cpd11	18.71	ACTIVE	n.a.
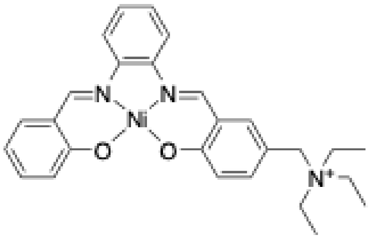	Cpd12	15.61	ACTIVE	n.a.
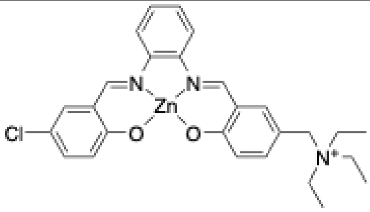	Cpd13	1.68	ACTIVE	n.a.

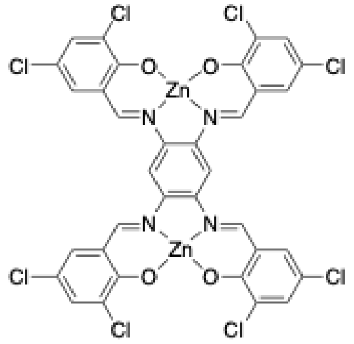	Cpd14	0.82	INACTIVE	n.a.
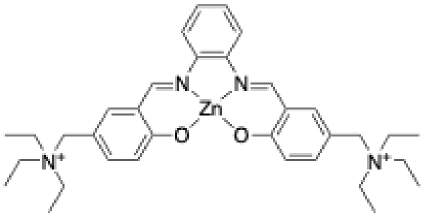	Cpd15	5.81	ACTIVE	1
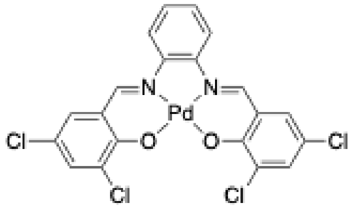	Cpd16	2.30	INACTIVE	2
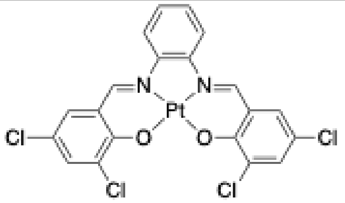	Cpd17	3.70	INACTIVE	2
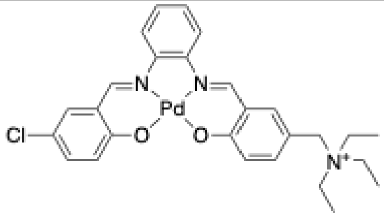	Cpd18	7.20	ACTIVE	n.a.
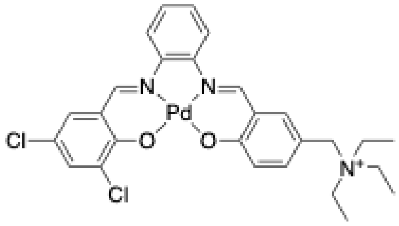	Cpd19	>25	ACTIVE	n.a.

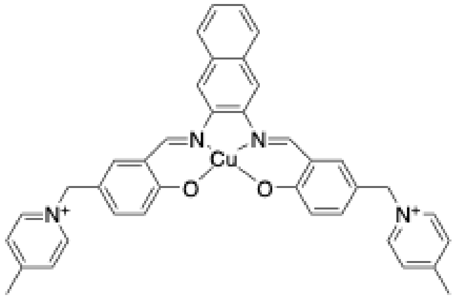	Cpd20	23.70	ACTIVE	3
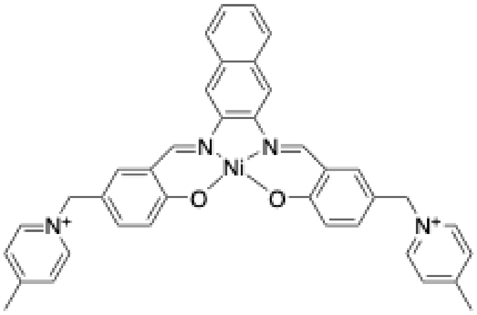	Cpd21	31.50	ACTIVE	3
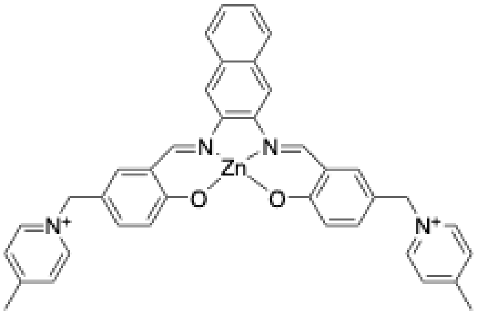	Cpd22	15.43	ACTIVE	3
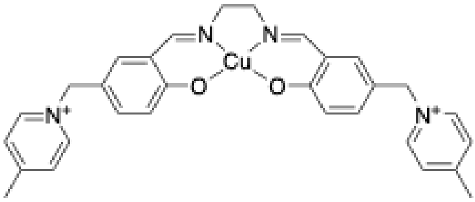	Cpd23	4.16	ACTIVE	3
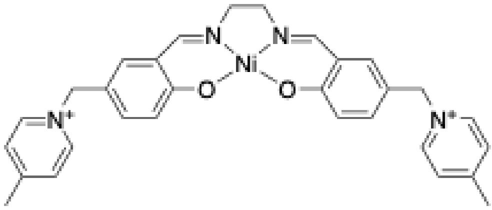	Cpd24	8.08	ACTIVE	3

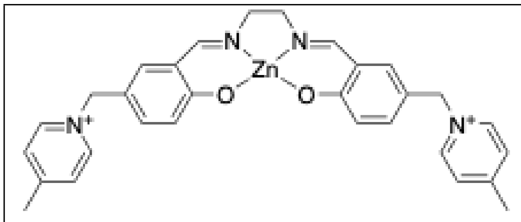	Cpd25	0.99	ACTIVE	3
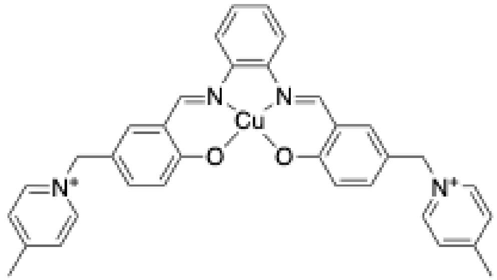	Cpd26	22.59	ACTIVE	3
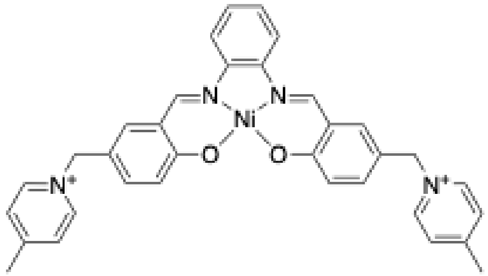	Cpd27	28.98	ACTIVE	3
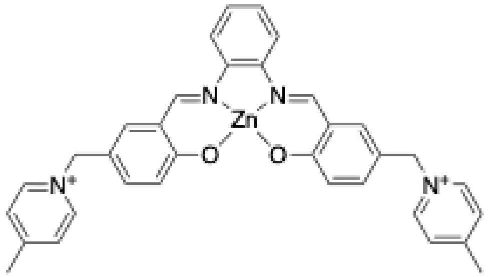	Cpd28	10.65	ACTIVE	3

References

(1) D'Anna, L.; Froux, A.; Bonsignore, R.; Roller, A.; Kowol, C. R.; Monari, A.; Grandemange, S.; Barone, G.; Terenzi, A. *In Medio Stat Virtus%*: Asymmetric Salphen Metal Complexes with Improved Biological Properties. *Dalton Trans.* **2026**, *55* (10), 4250–4258. https://doi.org/10.1039/D5DT02881G.

(2) D'Anna, L.; Rubino, S.; Pipitone, C.; Serio, G.; Gentile, C.; Palumbo Piccionello, A.; Giannici, F.; Barone, G.; Terenzi, A. Salphen Metal Complexes as Potential Anticancer Agents: Interaction Profile and Selectivity Studies toward the Three G-Quadruplex Units in the *KIT* Promoter. *Dalton Trans.* **2023**, *52* (10), 2966–2975. https://doi.org/10.1039/D2DT03229E.

(3) Froux, A.; D'Anna, L.; Rainot, A.; Neybecker, C.; Spinello, A.; Bonsignore, R.; Rouget, R.; Harlé, G.; Terenzi, A.; Monari, A.; Grandemange, S.; Barone, G. Metal Centers and Aromatic Moieties in

Schiff Base Complexes: Impact on G-Quadruplex Stabilization and Oncogene Downregulation. *Inorg. Chem. Front.* **2024**, *11* (17), 5725–5740. https://doi.org/10.1039/D4QI01394H.
